# Metabolic vulnerability index and MetaboHealth score as risk factors for age-related macular degeneration in a large-scale prospective cohort

**DOI:** 10.1186/s12967-026-07863-2

**Published:** 2026-02-18

**Authors:** Jun Yu, Yuzhou Zhang, Yue Lan Gao, Jian Ming Xu, Haoyu Chen, Mary Ho, Ka Wai Kam, Alvin L. Young, Chi Pui Pang, Clement C. Tham, Jason C. Yam, Li Jia Chen

**Affiliations:** 1 Department of Ophthalmology and Visual Sciences, The Chinese University of Hong Kong, Hong Kong SAR, China; 2 Joint Shantou International Eye Center of Shantou University and the Chinese University of Hong Kong, Guangdong, Shantou, China; 3 Department of Ophthalmology and Visual Sciences, Prince of Wales Hospital, Hong Kong SAR, China; 4 Hong Kong Eye Hospital, Hong Kong SAR, China

**Keywords:** Age-related macular degeneration, Metabolomics, Polygenic risk score, Retinal imaging

## Abstract

**Background:**

Age-related macular degeneration (AMD) imposes a substantial burden of disability. Metabolic disturbances are associated with increased AMD risk. Here we evaluate the metabolic vulnerability index (MVX), derived from inflammation- and malnutrition-related markers, and MetaboHealth score, developed from metabolomics-based biological aging, as biomarkers for AMD incidence.

**Methods:**

UK Biobank participants with metabolomic data and no AMD at baseline were included. Cox proportional hazards models were used to estimate the associations of MVX and MetaboHealth with incident AMD, adjusting for demographic, socioeconomic, lifestyle, and comorbidity factors. Interactions between AMD polygenic risk score (PRS, field 26,204) and both scores were evaluated on both multiplicative and additive scales using the relative excess risk due to interaction (RERI), attributable proportion (AP), and synergy index (SI). Cross-sectional linear models were adopted to assess the associations of both scores with AMD-related retinal traits, including photoreceptor segment (PS) and retinal pigment epithelium–Bruch’s membrane thicknesses.

**Results:**

Over a median follow-up of 13.66 years, AMD occurred in 5509 of 265,133 participants for MVX analysis and in 5498 of 264,352 participants for MetaboHealth analysis. Higher quintiles of MVX and the MetaboHealth score demonstrated a dose-dependent association with increased AMD risk (*P* for trends < 0.001), with hazard ratios (HRs) of 1.17 (95% CI 1.06–1.30) and 1.32 (1.20–1.45) for the highest quintiles, respectively. Both associations were stronger in those with specific comorbidities compared with their respective reference groups (multiplicative *p* < 0.05). The highest risk was observed for individuals with jointly high PRS and MVX or MetaboHealth score (HR 2.53 [2.06–3.10]; HR 3.14 [2.54–3.89], respectively). AMD PRS interacted with MVX on AMD risk (multiplicative *p* < 0.05, RERI = 0.27 [0.13–0.41], AP = 0.16 [0.08–0.23], and SI = 1.58 [1.10–2.05]). This multiplicative interaction was further supported by genetic variants in the *complement factor H* gene. Both higher MVX and MetaboHealth scores were inversely associated with PS thickness (β = −0.09 µm and β = −0.22 µm, both *p* < 0.05).

**Conclusions:**

MVX and the MetaboHealth score are associated with AMD risk, especially in individuals with comorbidities. Further studies are needed to elucidate the underlying mechanisms and assess potential causal relationships.

**Supplementary information:**

The online version contains supplementary material available at 10.1186/s12967-026-07863-2.

## Background

Age-related macular degeneration (AMD), a leading cause of blindness, affected 196 million people worldwide in 2020 and is projected to affect 288 million by 2040. It poses a major global public health challenge, especially in aging populations [1, 2]. AMD imposes a considerable personal, societal, and economic burden, with significant consequences for work capacity and quality of life. The biological mechanisms underlying AMD are complex, encompassing both genetic predisposition and a broad spectrum of environmental and lifestyle exposures accumulated throughout the lifespan [3, 4]. Identification of modifiable risk factors for AMD would aid in understanding its underlying mechanisms and developing targeted strategies for disease prevention.

The serum metabolites, which reflect the interplay of intrinsic physiology and external exposures, are increasingly recognized as valuable biomarkers for a broad spectrum of diseases and health outcomes [5]. Recent research has progressed from phenotypic associations between AMD and systemic diseases [6, 7], to linking AMD with metabolic processes. This underscores the potential role of circulating metabolites as a connection between AMD and systemic health [8–15]. However, these findings were largely exploratory and fragmented, based on individual metabolites and involved heterogeneous sets of metabolites across studies, and have yet to converge on clinically translatable metrics. The metabolic vulnerability index (MVX), a recently developed metric, is derived from six metabolites quantified by a clinically implemented nuclear magnetic resonance (NMR) blood test, capturing biological dimensions of metabolic malnutrition–inflammation syndromes [16]. Recent hospital-based studies have also demonstrated strong associations of MVX with patient survival in cardiovascular populations, highlighting its role as a biomarker of systemic metabolic vulnerability [16, 17]. In addition, MetaboHealth, a novel metabolomics-based aging score developed from mortality outcomes, has been illustrated as a promising marker for mortality risk with potential clinical application [18]. Owing to their standardized measurements and ease of implementation, both MVX and MetaboHealth provide promising bases for clinical translation. To date, however, whether these two indices can serve as risk indicators for AMD incidence has not been examined.

To address these research and clinical gaps, we conducted a prospective longitudinal analysis to investigate the association of MVX and MetaboHealth with AMD incidence using data from the UK Biobank (UKB). We further examined whether genetic predisposition to AMD modifies these associations on both additive and multiplicative scales. Finally, we assessed two AMD-related retinal traits—the photoreceptor segment layer (PS) and the retinal pigment epithelium–Bruch’s membrane (RPE–BM) complex—to provide structural evidence for these associations[1, 19, 20].

## Methods

### Study participants

The UKB study protocol is publicly available (https://www.ukbiobank.ac.uk/). Between 2006 and 2010, over half a million participants aged 40–69 years were recruited from 22 assessment centers nationwide, where comprehensive baseline data were collected through standardized sociodemographic questionnaires, physical and functional measurements, and biospecimen sampling [21]. The UKB has ethical approval from the North West Multi-Centre Research Ethics Committee (REC reference: 21/NW/0157) and the National Information Governance Board for Health and Social Care. All procedures adhered to the principles of the Declaration of Helsinki.

### Metabolomic profiles and metabolic vulnerability index calculation

Plasma metabolite levels in the UKB were profiled with the Nightingale Health (Finland) high-throughput NMR spectroscopy platform. In the present study, we adopted the Phase 2 release of the metabolomics data, which includes approximately 275,000 participants with baseline measurements. Detailed protocols of the technical methodology are available at (https://biobank.ndph.ox.ac.uk/showcase/ukb/docs/NMR_companion_phase2.pdf). In brief, for laboratory processing, cryopreserved plasma was thawed, centrifuged to remove precipitates, and the resulting supernatant was mixed with a phosphate buffer. The prepared samples were loaded into a cooled autosampler, and two spectra per plasma sample were acquired on a 500 MHz NMR spectrometer (Bruker).

The sex-specific MVX score was constructed to quantify the intertwined metabolic malnutrition–inflammation syndrome, using coefficients derived from predictive models for all-cause mortality applied to six mortality-associated biomarkers in cardiovascular cohorts [16]. It includes glycoprotein acetyls (GlycA), small high-density lipoprotein particles (sHDL), citrate, and three branched-chain amino acids (BCAAs): isoleucine, leucine and valine, and was then scaled to a 1–100 range following the published protocol [16]. The full MVX equation and its two components, the inflammation vulnerability index (IVX) and the metabolic malnutrition index (MMX), are provided in Table S1 (see Additional file 1).

MetaboHealth score was developed as a weighted sum of 14 log-transformed NMR biomarkers using coefficients derived from external cohorts, capturing a metabolic signature of long-term all-cause mortality risk and serving as a broader proxy for biological aging [18, 22]. The included biomarkers comprised albumin, GlycA, glucose, lactate, histidine, isoleucine, leucine, valine, phenylalanine, acetoacetate, and selected lipid measures (VLDL particle diameter, total lipids in chylomicrons and extremely large VLDL, total lipids in small HDL, and the ratio of polyunsaturated to total fatty acids). Four metabolites (GlycA, isoleucine, leucine, and valine) in the MetaboHealth score overlap with those included in the MVX. Both scores were calculated at the individual participant level by applying the respective formulas among participants with complete data for the corresponding metric, with higher values indicating greater metabolic disturbance. All metrics were converted to z-scores before analysis to facilitate direct comparison [23].

### Definition of AMD

Diagnostic information was linked with the national Hospital Episode Statistics (HES), self-report, and death registry data [24]. AMD was identified using multiple data sources in the UKB. First, hospital records were screened for AMD diagnoses based on the International Classification of Diseases (ICD), including ICD-10 code H35.3 (fields 41,202 and 41,204) and ICD-9 code 3625 (fields 41,203 and 41,205). The date of the earliest hospital admission diagnosis was obtained through linkage with HES data (fields 41,281 and 41,280). Second, self-reported information was incorporated, including macular degeneration (field 20,002; code 1528) and the interpolated age at first diagnosis (field 20,009). Third, ophthalmic examination data were used (category 100,041), comprising laterality of the affected eye (field 5912), responses to the survey on eye disorders (field 6148), and reported age at diagnosis (field 5923).

Prevalent AMD was defined as a diagnosis documented before baseline assessment or within six months thereafter. Incident AMD was defined as the first diagnosis occurring more than six months after recruitment. Hospital admission records were available until 31 October 2022; therefore, follow-up was censored at the earliest occurrence of AMD diagnosis, death, or the end of data availability (31 October 2022).

### Retinal layer assessment

With the widespread use of risk-free optical coherence tomography (OCT) in clinical practice, thickening of RPE–BM complex and thinning of PS layer have been recognized as early hallmarks of AMD pathophysiology [1, 19, 20]. During the baseline assessment period (2009–2010), a subset of approximately 65,000 participants received OCT imaging performed with a Topcon 3D OCT1000 Mark II device [25, 26]. Retinal layer segmentation was performed using the Topcon Advanced Boundary Segmentation (TABS) algorithm (version 1.6.1.1). Details of the image quality control protocol have been published previously [27]. In brief, scans with signal strength below 45 or showing major segmentation errors, including pronounced motion artifacts, clipping, or edge detection failure, were excluded to ensure that only images of sufficient quality were retained (see Additional file 1: Methods) [28]. For each participant, retinal layer thickness was averaged across both eyes when data from both eyes were available. If only one eye passed quality control, the thickness value from that eye was used.

### Statistical analysis

R (version 4.2.2) was used for all statistical analyses. Continuous variables are reported as mean (standard deviation [SD]), and categorical variables as number (percentage).

The prospective association between each metabolic metric and AMD incidence was assessed using multivariable Cox proportional hazards (CPH) regression models. Model 1 was adjusted for age and sex. Model 2 was additionally adjusted for self-reported ethnicity, smoking status, education, Townsend Deprivation Index, body mass index (BMI), and comorbidities including cardiovascular disease (CVD), hypertension, diabetes, and chronic kidney disease (CKD) (Additional file 1: Methods and Table S2) [8, 20]. Missing values for each confounder variable were less than 2%. The proportional hazards assumption, assessed with Schoenfeld residuals, was satisfied. The correlation matrix covering all study variables is shown in Figure S1 (see Additional file 1). Both MVX and the MetaboHealth score were modeled as continuous variables (per 1-SD increment) and as quintiles, using the lowest quintile as the reference. Linear trends were tested by assigning the median value of each quintile and modeling it as a continuous variable. Cumulative incidence curves were plotted to compare the cumulative incidence of AMD across quintiles of the metabolic metrics (Q1, Q2–Q4, and Q5). The primary exposures were MVX and MetaboHealth score, with IVX and MMX, intermediate indices contributing to MVX, additionally examined for their independent associations.

To test the robustness of our findings, we performed several sensitivity analyses for addressing potential sources of bias, including (i) adjustment for HDL, total cholesterol, triglycerides and lipid-lowering medication to account for residual lipid-related confounding; (ii) performing a 1:1 age- and sex-matched propensity score analysis (caliper = 0.01) and re-fitted CPH models with adjustment for all other covariates listed above [29], (iii) restricting the outcome definition to ICD-coded AMD to minimize potential misclassification; and (iv) applying a 10-year follow-up window to examine the medium-term stability of associations.

To assess the incremental value of MVX and MetaboHealth beyond established AMD risk factors, we compared model discrimination between a base Cox model including conventional clinical covariates (all covariates in Model 2) and models additionally including MVX or MetaboHealth. Model discrimination was quantified using the Harrell’s C-statistic. Differences in the C-statistics were calculated to evaluate incremental improvements in discrimination.

Subgroup analyses were conducted across age, smoking status, and major systemic conditions, which are well-established risk factors for AMD, with interaction tests used to assess whether these factors modified the associations of MVX and MetaboHealth with AMD. Since AMD has a strong genetic basis [4], we subsequently performed interaction analyses to test whether the polygenic risk score (PRS) for AMD would modify the associations of MVX and MetaboHealth score with AMD incidence among individuals of European ancestry. The PRS for AMD (data field 26,204 in UKB), using summary statistics from an external GWAS meta-analysis, was variance-standardized [30] and has shown good performance for AMD prediction [31, 32]. The interaction was evaluated on two scales. Multiplicative interaction was assessed by including an interaction term in the CPH models, and significance was evaluated using the Wald test, while additive interaction (see Additional file 1: Methods) was estimated using measures such as the relative excess risk due to interaction (RERI), the attributable proportion (AP), and the synergy index (SI) [33, 34]. Since c*omplement factor H* (*CFH*) and *age-related maculopathy susceptibility 2* (*ARMS2*) are known genes for AMD [35, 36], we selected representative variants, namely rs1061170 and rs10922109 in *CFH* and rs3750846 and rs10490924 in *ARMS2*, to further illustrate their potential modifier effects.

The cross-sectional associations of PS layer and RPE–BM complex thickness with MVX were evaluated separately using multivariable linear regression models, adjusted for the covariates described above, as well as lipid-lowering medication use and alcohol consumption frequency [37]. In sensitivity analyses, additional adjustment was made for spherical equivalent (SE) and intraocular pressure (IOP).[37]

For the primary outcome, statistical significance was defined as a two-sided *p* < 0.05. For secondary retinal-layer outcomes, multiple comparisons were controlled using the false discovery rate (FDR), with FDR-adjusted *p* < 0.05 indicating statistical significance.

## Results

### Participant characteristics

**Fig. 1:**
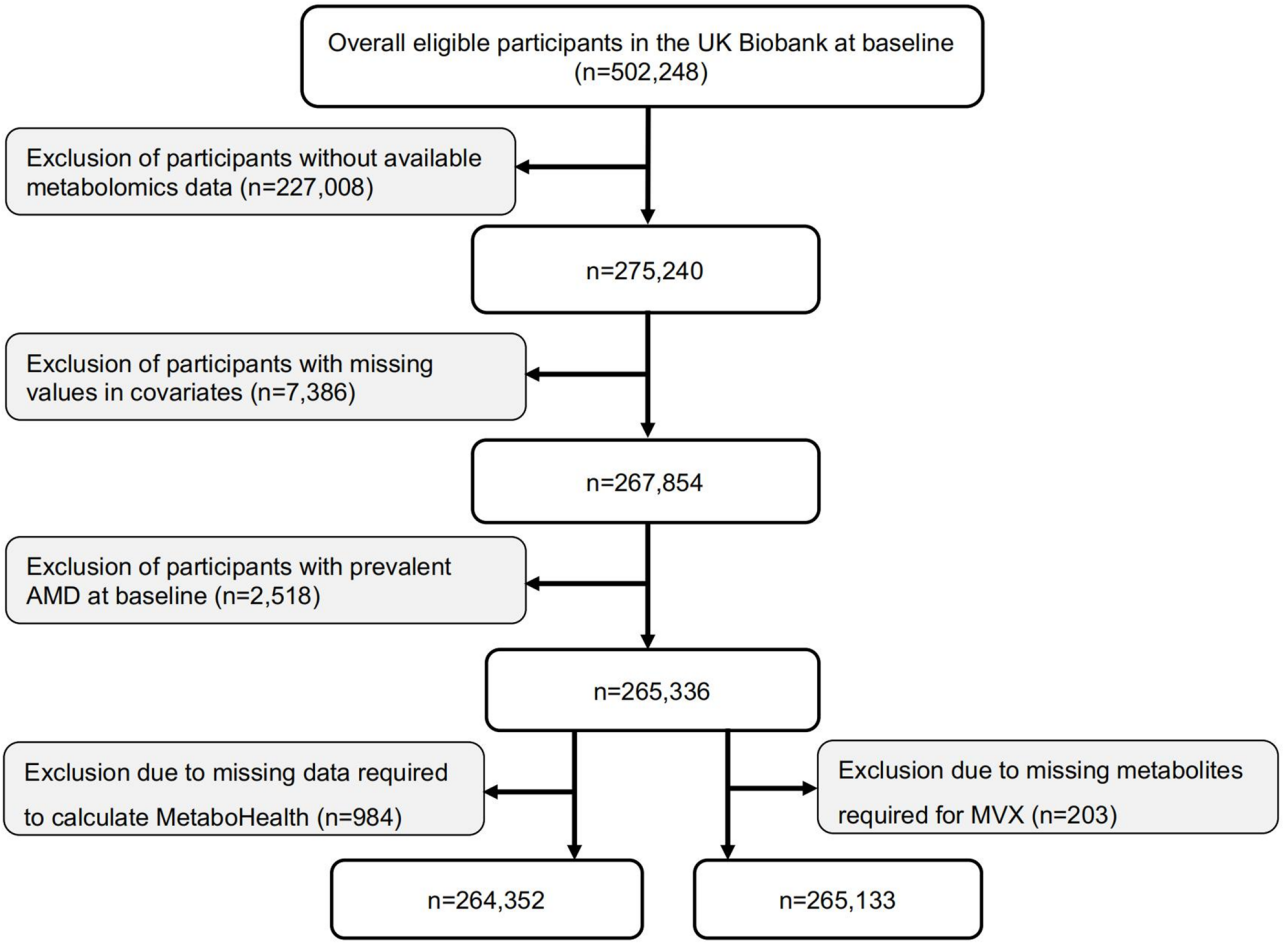
Flowchart of participant selection for prospective analysis. MVX, metabolic vulnerability index; AMD, age-related macular degeneration

We analyzed data for two overlapping groups of participants (Fig. 1). In brief, among 275,240 participants with metabolomics measurements, we excluded those who had missing covariates (*n* = 7,386), documented AMD at baseline (*n* = 2,518), and incomplete metabolite data required for score calculation. The final analytic samples included 265,133 participants for MVX and 264,352 for MetaboHealth analyses. Among participants for the MVX analysis, 54.0% were female, and the mean age was 56.5 (SD 8.07) years (Table 1). Participants with higher MVX values were more likely to be older, female, socioeconomically deprived, smokers, obese, and were less likely to have higher educational attainment. Similar patterns were observed among participants with a higher MetaboHealth score (Additional file 1: Table S3).

**Table 1:** Characteristics of participants at the baseline assessment by MVX quintile

Baseline characteristics	Whole cohort	Quintile, Q1	Quintile, Q2	Quintile, Q3	Quintile, Q4	Quintile, Q5
Number	265,133	53,027	53,026	53,027	53,026	53,027
Age, years, mean (SD)	56.50 (8.07)	56.17 (8.34)	56.24 (8.13)	56.19 (8.02)	56.63 (7.99)	57.24 (7.84)
Female, n (%)	143,244 (54.0)	5719 (10.8)	18,792 (35.4)	33,019 (62.3)	41,478 (78.2)	44,236 (83.4)
White, n (%)	252,648 (95.3)	50,069 (94.4)	50,590 (95.4)	50,734 (95.7)	50,607 (95.4)	50,648 (95.5)
TDI, mean (SD)	−1.38 (3.06)	−1.52 (3.02)	−1.48 (3.03)	−1.46 (3.00)	−1.37 (3.04)	−1.08 (3.17)
University degree, n (%)	84,500 (31.9)	20,736 (39.1)	18,280 (34.5)	17,137 (32.3)	15,483 (29.2)	12,864 (24.3)
Ever smoker, n (%)	120,178 (45.3)	23,863 (45.0)	24,897 (47.0)	23,675 (44.6)	23,107 (43.6)	24,636 (46.5)
BMI, mean (SD)	27.44 (4.78)	26.75 (4.01)	27.07 (4.36)	27.10 (4.58)	27.52 (4.96)	28.75 (5.57)
CVD history, n (%)	17,942 (6.8)	5075 (9.6)	3931 (7.4)	2939 (5.5)	2763 (5.2)	3234 (6.1)
Hypertension history, n (%)	78,867 (29.7)	15,272 (28.8)	15,409 (29.1)	14,717 (27.8)	15,175 (28.6)	18,294 (34.5)
CKD history, n (%)	3673 (1.4)	585 (1.1)	678 (1.3)	646 (1.2)	721 (1.4)	1043 (2.0)
Diabetes history, n (%)	14370 (5.4)	3269 (6.2)	2934 (5.5)	2456 (4.6)	2411 (4.5)	3300 (6.2)
IVX score, mean (SD)	0.00 (1.00)	−0.47 (0.89)	−0.24 (0.88)	−0.04 (0.90)	0.19 (0.93)	0.57 (1.06)
MMX score, mean (SD)	0.00 (1.00)	−0.07 (0.82)	−0.04 (0.95)	−0.00 (1.01)	0.02 (1.07)	0.09 (1.12)
MVX score, mean (SD)	0.00 (1.00)	−0.54 (0.92)	−0.28 (0.87)	−0.04 (0.87)	0.22 (0.88)	0.64 (1.01)
sHDL, µmol/L, mean (SD)	9.78 (1.34)	9.15 (1.17)	9.72 (1.15)	9.92 (1.19)	9.93 (1.29)	10.21 (1.59)
GlycA, µmol/L, mean (SD)	811.58 (120.26)	703.95 (66.21)	778.16 (74.54)	804.00 (89.59)	834.29 (98.11)	938.36 (124.52)
Citrate, µmol/L, mean (SD)	65.38 (13.11)	61.58 (12.46)	64.75 (12.44)	66.03 (12.83)	66.55 (13.01)	68.01 (13.86)
Isoleucine, µmol/L, mean (SD)	51.16 (18.23)	51.84 (15.38)	53.51 (17.28)	51.65 (17.69)	48.95 (18.35)	49.85 (21.56)
Valine, µmol/L, mean (SD)	210.31 (43.51)	211.81 (35.10)	216.16 (38.25)	211.87 (40.45)	204.05 (44.69)	207.62 (55.30)
Leucine, µmol/L, mean (SD)	104.18 (28.92)	109.77 (24.03)	109.11 (26.43)	104.83 (27.14)	98.81 (28.91)	98.32 (34.88)

BMI, body mass index; TDI, Townsend Deprivation Index; CVD, cardiovascular disease; CKD, chronic kidney disease; IVX, inflammation vulnerability index; MMX, metabolic malnutrition index; MVX, metabolic vulnerability index; sHDL, small high-density lipoprotein particles; GlycA, glycoprotein acetyls

### Prospective association between metabolic metrics and AMD incidence

**Fig. 2:**
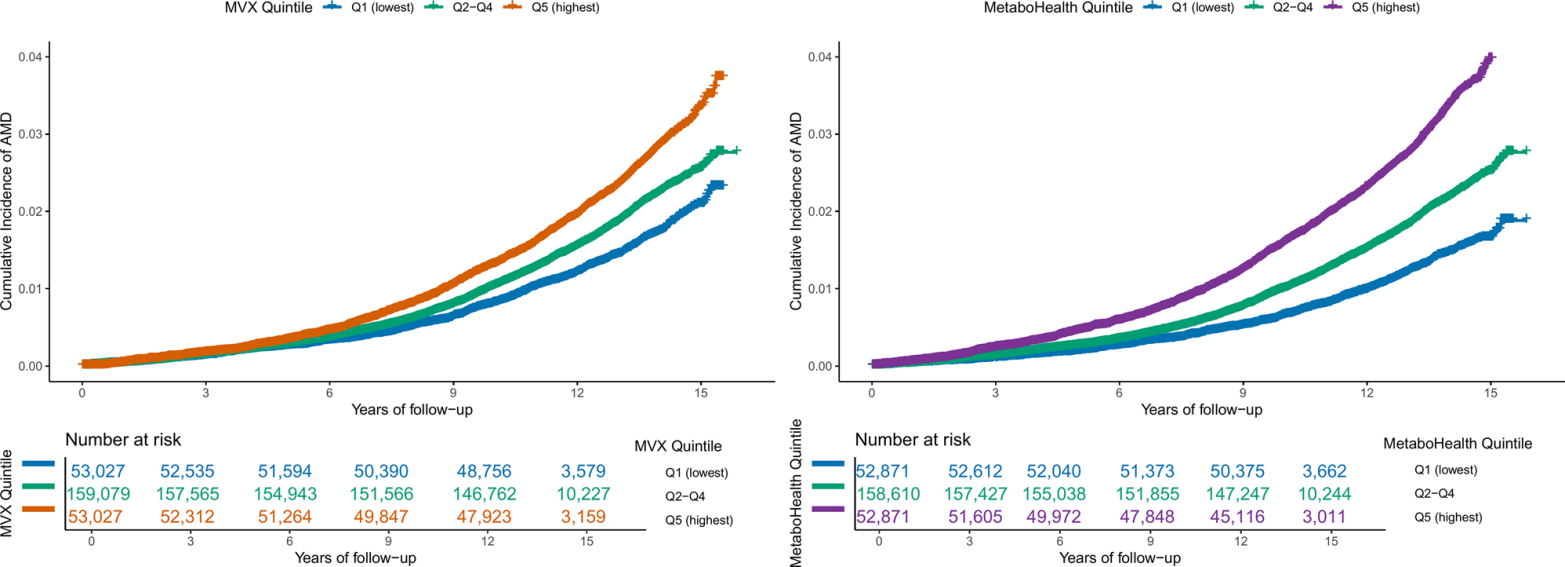
Cumulative incidence curves for age-related macular degeneration. (**A**) cumulative incidence of AMD by MVX quintiles. (**B**) cumulative incidence of AMD by MetaboHealth quintiles. Quintiles are grouped as Q1 (lowest), Q2–Q4 (middle), and Q5 (highest). Curves represent AMD cumulative incidence estimated over follow-up time. Numbers at risk are shown below each panel. MVX, metabolic vulnerability index

During a median follow-up of 13.66 years, 5509 (2.08%) participants in the MVX analysis and 5498 (2.08%) participants in the MetaboHealth analysis were newly diagnosed with AMD. MVX and MetaboHealth were moderately correlated (Pearson *r* = 0.46; Additional file 1: Figure S1). As shown in Fig. 2, participants with higher MVX and the MetaboHealth score had a higher cumulative incidence of AMD, with the highest quintile consistently showing the greatest incidence compared with the lowest quintile (*p* < 0.0001).

Both age- and sex-adjusted (Model 1) and fully adjusted (Model 2) models showed consistent associations of higher MVX and MetaboHealth scores with increased AMD incidence (Table 2). In Model 2, each SD increase in the MVX score was associated with a 6% higher risk of AMD (HR, 1.06; 95% CI, 1.02–1.09), and each SD increase in the MetaboHealth score was associated with a 14% higher risk (HR, 1.14; 95% CI, 1.11–1.17). Similar associations with AMD incidence were observed for the MMX (HR, 1.06; 95% CI, 1.03–1.09) and IVX (HR, 1.04; 95% CI, 1.00–1.07). In quintile analyses, participants in the highest versus lowest quintile had an increased risk of AMD, with a 17% increase for MVX (HR, 1.17; 95% CI, 1.06–1.30; *P* for trend < 0.001) and a 32% increase for MetaboHealth (HR, 1.32; 95% CI, 1.20–1.45; *P* for trend < 0.001). Four sensitivity analyses yielded consistent results for the main associations (Additional file 1: Tables S4–S5).

**Table 2:** Associations of baseline MVX, IVX, MMX, and MetaboHealth with incident age-related macular degeneration

	HR (95% CI)	P	Quintile, Q1	Quintile, Q2	Quintile, Q3	Quintile, Q4	Quintile, Q5	P for trend
**MVX**								
Event/N	5509/265,133	NA	857/53,027	991/53,026	1079/53,027	1214/53,026	1368/53,027	NA
Age and sex adjusted	1.10 (1.07–1.13)	<0.001	1.00 (ref)	1.11 (1.02–1.22)	1.14 (1.03–1.26)	1.18 (1.07–1.30)	1.27 (1.15–1.41)	<0.001
Fully adjusted	1.06 (1.02–1.09)	<0.001	1.00 (ref)	1.09 (1.00–1.20)	1.10 (1.00–1.21)	1.11 (1.01–1.23)	1.17 (1.06–1.30)	<0.001
**MMX**								
Event/N	5509/265,133	NA	1233/53,118	1150/53,071	960/53,086	1040/53,011	1126/52,847	NA
Age and sex adjusted	1.04 (1.01–1.07)	0.01	1.00 (ref)	1.06 (0.97–1.14)	1.04 (0.95–1.14)	1.09 (0.99–1.20)	1.13 (1.03–1.23)	0.007
Fully adjusted	1.06 (1.03–1.09)	<0.001	1.00 (ref)	1.09 (1.01–1.19)	1.09 (0.99–1.20)	1.15 (1.05–1.27)	1.20 (1.10–1.32)	<0.001
**IVX**								
Event/N	5509/265,133	NA	910/52,977	910/53,129	1025/53,198	1240/53,071	1424/52,758	NA
Age and sex adjusted	1.09 (1.05–1.12)	<0.001	1.00 (ref)	1.04 (0.95–1.14)	1.11 (1.00–1.22)	1.15 (1.04–1.28)	1.23 (1.10–1.37)	<0.001
Fully adjusted	1.04 (1.00–1.07)	0.04	1.00 (ref)	1.01 (0.92–1.11)	1.06 (0.96–1.18)	1.07 (0.96–1.19)	1.09 (0.98–1.22)	0.22
**MetaboHealth**	NA	NA						
Event/N	5498/264,352	NA	726/52,871	887/52,870	1077/52,870	1243/52,870	1565/52,871	NA
Age and sex adjusted	1.19 (1.16–1.21)	<0.001	1.00 (ref)	1.01 (0.91–1.11)	1.11 (1.01–1.22)	1.19 (1.08–1.30)	1.42 (1.29–1.55)	<0.001
Fully adjusted	1.14 (1.11–1.17)	<0.001	1.00 (ref)	1.00 (0.90–1.10)	1.08 (0.98–1.19)	1.13 (1.03–1.25)	1.32 (1.20–1.45)	<0.001

Full models adjusted for: age, sex, ethnic background, smoking, education level, Townsend deprivation index, body mass index, history of cardiovascular disease, history of hypertension, history of chronic kidney disease, and history of diabetes. MVX metabolic vulnerability index; MMX, metabolic malnutrition index; IVX, inflammation vulnerability index; HR, hazard ratio; SD, standard deviation

### Model discrimination analyses

In discriminative performance analyses, the conventional clinical model incorporating the MetaboHealth score achieved a C statistic of 0.752 (95% CI 0.747–0.758) and demonstrated an additional discriminatory value (ΔC = 0.0022, 95% CI 0.0012–0.0031; *p* < 0.001), whereas the inclusion of MVX did not improve model discrimination (Additional file 1: Table S6).

### Subgroup analyses

Subgroup analyses for MVX yielded findings comparable to the main results when modeled continuously (Additional file 1: Tables S7–S8). Participants with CVD had a 16% higher risk of incident AMD (HR, 1.16; 95% CI, 1.06–1.27; *p* = 0.0016), whereas those without CVD had a 5% higher hazard (HR, 1.05; 95% CI, 1.01–1.08; *p* = 0.008; multiplicative *p* < 0.05). Similar patterns were observed in participants with or without other comorbidities, including CKD (HR, 1.24; 95% CI, 1.02–1.50 vs. HR, 1.06; 95% CI, 1.02–1.09), hypertension (HR, 1.08; 95% CI, 1.03–1.13 vs. HR, 1.05; 95% CI, 1.01–1.09), and diabetes (HR, 1.11; 95% CI, 1.01–1.22 vs. HR, 1.06; 95% CI, 1.02–1.09), although significant interactions were not observed. There was no evidence of interaction with age or smoking status, with similar MVX associations in older and younger individuals, and in never and ever smokers.

Subgroup analyses of the MetaboHealth score showed results broadly consistent with the main findings (Additional file 1: Tables S9–S10). Notably, an interaction with age was observed on the additive scale, with older participants showing greater combined effects (RERI = 0.81, 95% CI, 0.45–1.17; AP = 0.13, 95% CI, 0.07–0.19; SI = 1.19, 95% CI, 1.09–1.28), but not on the multiplicative scale (multiplicative *p* = 0.05). For comorbidities, the association between MetaboHealth and AMD was stronger among participants with diabetes than among those without; the highest quintile of the MetaboHealth score was associated with a 60% higher risk of AMD (HR, 1.60; 95% CI, 1.11–2.30) in diabetic individuals, compared with a 22% higher risk (HR, 1.22; 95% CI, 1.10–1.34) in non-diabetic participants (multiplicative *p* < 0.05). The associations between MetaboHealth and AMD incidence were similar across subgroups stratified by other comorbidities: CKD, hypertension, and CVD (all multiplicative *p* > 0.05).

### Interaction between metabolic profiles and genetic risk for AMD during follow-up

**Fig. 3:**
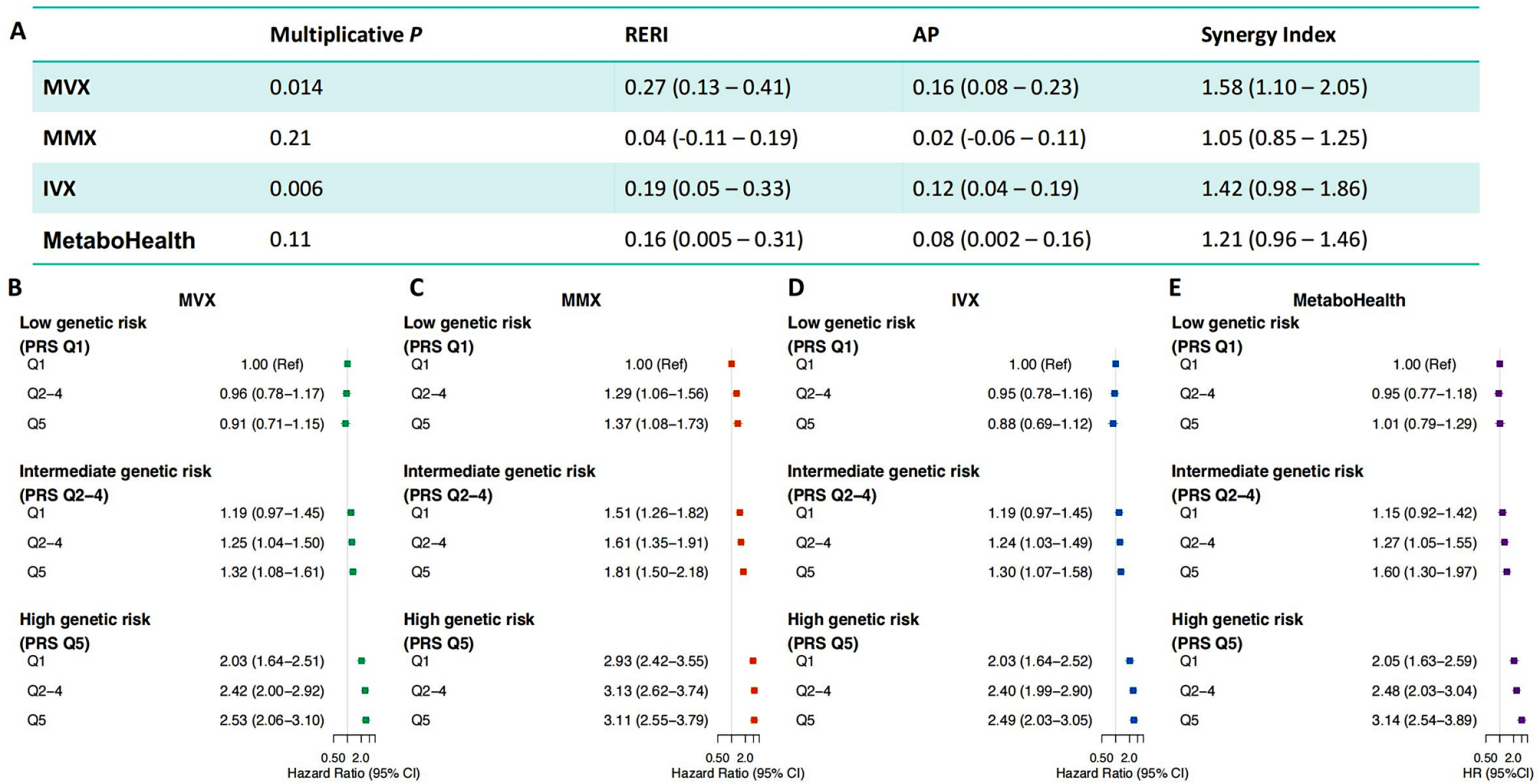
Additive and multiplicative interactions between metabolic metrics and polygenic risk score on the risk of incident AMD in European participants. (**A**) multiplicative and additive interaction estimates between each metabolic metric and the AMD polygenic risk score (PRS). (**B**–**E**) joint associations of MVX, MMX, IVX, and MetaboHealth score with PRS for incident AMD. Participants were stratified by PRS quintiles and metabolic metric quintiles (Q1, lowest; Q2–Q4, intermediate; Q5, highest), with hazard ratios (HRs) and 95% confidence intervals (CIs) for incident AMD estimated for each combination, using the lowest metabolic quintile (Q1) and lowest PRS group as the reference. All models were adjusted for age, sex, smoking, education level, Townsend deprivation index, body mass index, history of cardiovascular disease, history of hypertension, history of chronic kidney disease, and history of diabetes. MVX, metabolic vulnerability index; MMX, metabolic malnutrition index; IVX, inflammation vulnerability index; RERI, relative excess risk due to interaction; AP, attributable proportion; SI, synergy index

Figure 3 Ademonstrates significant multiplicative interactions between MVX and AMD PRS (*p* = 0.014) and between IVX and AMD PRS (*p* = 0.006) in participants of European ancestry. Notably, a positive additive effect was also detected for MVX (RERI = 0.27, 95% CI, 0.13–0.41; AP = 0.16, 95% CI, 0.08–0.23; SI = 1.58, 95% CI, 1.10–2.05). By contrast, no significant multiplicative interactions were observed for MMX or MetaboHealth.

To better illustrate the interaction on the risk scale, we evaluated the joint effects by combining these metrics with the PRS (Fig. 3B–E). Participants in the highest quintile of both MVX and PRS had the greatest risk of incident AMD (HR, 2.53; 95% CI, 2.06–3.10), with a similar joint effect between PRS and IVX (HR, 2.49; 95% CI, 2.03–3.05) and MetaboHealth score (HR, 3.14; 95% CI, 2.54–3.89), whereas no comparable increasing trend was observed for MMX.

**Fig. 4:**
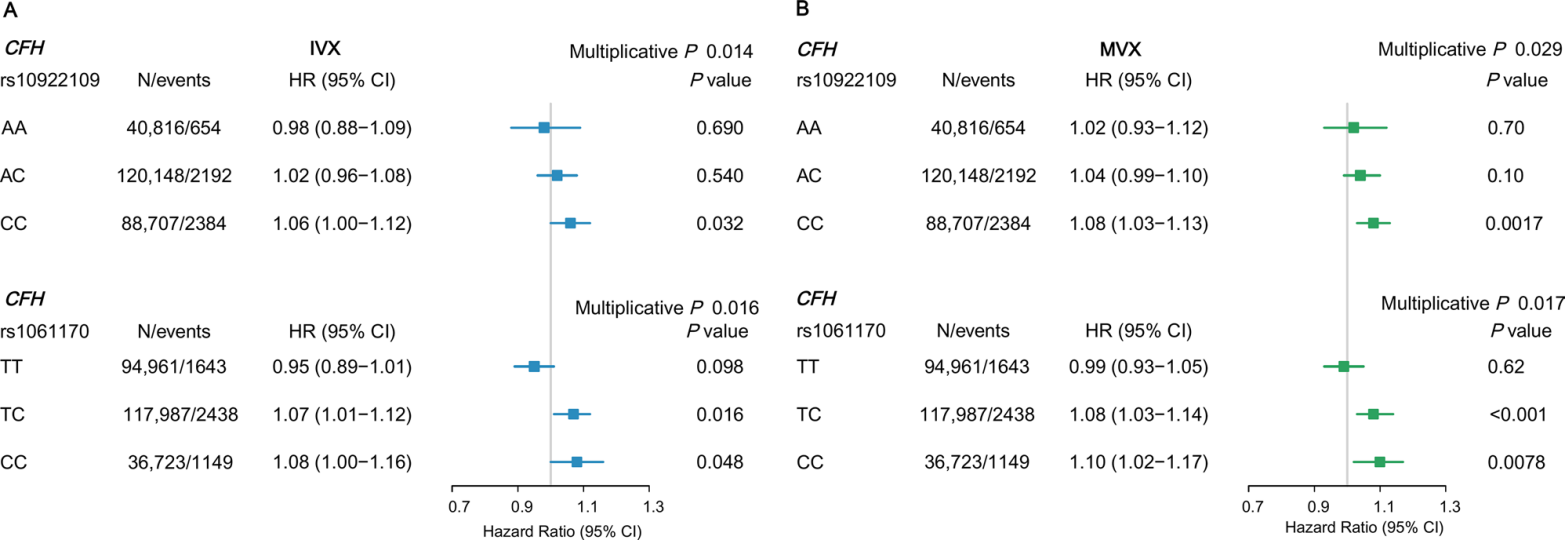
Forest plot showing IVX and MVX associations with incident AMD stratified by *CFH* genotypes in European participants. Hazard ratios (HRs) and 95% confidence intervals (CIs) are shown per 1-standard deviation increase in (**A**) IVX and (**B**) MVX, stratified by *CFH* rs10922109 and rs1061170 genotypes. All models were adjusted for age, sex, smoking, education level, Townsend deprivation index, body mass index, history of cardiovascular disease, history of hypertension, history of chronic kidney disease, and history of diabetes. MVX, metabolic vulnerability index; IVX, inflammation vulnerability index

Figure 4 illustrates the interactions between *CFH* variants (rs10922109 and rs1061170) and IVX and MVX on AMD risk (both multiplicative *p* < 0.05). For *CFH* rs10922109, higher MVX was associated with an increased AMD risk in participants with the homozygous CC allele (HR, 1.08; 95% CI, 1.03–1.13; *p* = 0.0017), while no significant association was observed in AA or AC carriers. For *CFH* rs1061170, significant associations were observed in participants with genotypes TC (HR, 1.08; 95% CI, 1.03–1.14; *p* < 0.001) and CC (HR, 1.10; 95% CI, 1.02–1.17; *p* = 0.0078), but not in those with TT homozygotes (*p* = 0.62). In contrast, no significant interactions were observed between *ARMS2* genotypes and MVX for AMD risk (Additional file 1: Table S11), nor between *CFH* or *ARMS2* variants and MetaboHealth score (Additional file 1: Table S12).

### Cross-sectional association between metabolic metrics and retinal layer thickness

Of 67,321 participants with baseline retinal images, 35,921 remained after quality control. By excluding participants with missing covariates or required metabolites, the cross-sectional analytic samples comprised 19,377 participants for MetaboHealth and 19,422 for MVX analyses (Additional file 1: Figure S2). Table S13 summarizes the baseline characteristics of participants for the retinal layer analyses (see Additional file 1). In fully adjusted models, higher MVX (per 1-SD increment) was associated with reduced PS layer thickness (β = −0.09 µm; 95% CI, −0.17 to −0.02; FDR-adjusted *p* = 0.035), but not with RPE–BM complex thickness (β = −0.05 µm; 95% CI, −0.09 to 0.00; FDR-adjusted *p* = 0.10; Table 3). For the MetaboHealth score, each 1-SD increment was inversely associated with PS thickness (β, −0.22 µm, 95% CI: −0.28 to −0.15; FDR-adjusted *p* < 0.001), with a pronounced effect in the top quintile (Q5 vs. Q1: β = −0.45 µm, 95% CI, −0.66 to −0.25; *P* for trend < 0.001). No association was detected for RPE–BM complex thickness. Associations of MVX and MetaboHealth with each PS sublayer are presented in Table S14 (see Additional file 1), and the results remained robust in sensitivity analyses (Additional file 1: Table S15).

**Table 3:** Associations of MVX, IVX, MMX, and MetaboHealth with baseline retinal layer thickness

Metric		PS layer thickness			**RPE**–**BM complex thickness**		
		Beta (95% CI)	P	FDR P	Beta (95% CI)	P	FDR P
**MVX**	Per SD	−0.09 (−0.17, −0.02)	0.01	0.035	−0.05 (−0.09, 0.00)	0.05	0.10
	Q1	ref	NA		ref	NA	
	Q2	0.11 (−0.12, 0.35)	0.34		−0.06 (−0.18, 0.06)	0.30	
	Q3	0.10 (−0.12, 0.32)	0.37		−0.05 (−0.18, 0.08)	0.42	
	Q4	−0.02 (−0.24, 0.20)	0.85		−0.14 (−0.28, −0.01)	0.036	
	Q5	−0.02 (−0.23, 0.20)	0.88		−0.16 (−0.31, −0.02)	0.025	
*P for trend*		0.03	NA		0.015	NA	
**MMX**	Per SD	−0.01 (−0.08, 0.05)	0.67	0.71	−0.03 (−0.08, 0.01)	0.12	0.19
	Q1	ref	NA		ref	NA	
	Q2	0.12 (−0.12, 0.36)	0.32		−0.08 (−0.23, 0.06)	0.27	
	Q3	0.13 (−0.09, 0.35)	0.25		−0.11 (−0.25, 0.03)	0.12	
	Q4	0.00 (−0.22, 0.22)	1.00		−0.06 (−0.20, 0.08)	0.38	
	Q5	−0.02 (−0.23, 0.20)	0.89		−0.11 (−0.24, 0.02)	0.11	
*P for trend*		0.51	NA		0.22	NA	
**IVX**	Per SD	−0.11 (−0.19, −0.02)	0.013	0.035	−0.04 (−0.09, 0.01)	0.15	0.20
	Q1	ref	NA		ref	NA	
	Q2	0.08 (−0.11, 0.28)	0.39		−0.11 (−0.23, 0.01)	0.066	
	Q3	−0.06 (−0.28, 0.15)	0.56		−0.07 (−0.20, 0.07)	0.33	
	Q4	−0.08 (−0.31, 0.15)	0.50		−0.09 (−0.23, 0.06)	0.25	
	Q5	−0.28 (−0.53, −0.03)	0.026		−0.13 (−0.28, 0.03)	0.11	
*P for trend*		0.014	NA		0.22	NA	
**MetaboHealth**	Per SD	−0.22 (−0.28, −0.15)	<0.001	<0.001	−0.01 (−0.05, 0.03)	0.71	0.71
	Q1	ref	NA		ref	NA	
	Q2	0.06 (−0.13, 0.26)	0.52		−0.02 (−0.14, 0.10)	0.73	
	Q3	−0.25 (−0.45, −0.05)	0.013		−0.02 (−0.15, 0.10)	0.72	
	Q4	−0.35 (−0.55, −0.15)	<0.001		−0.03 (−0.16, 0.09)	0.60	
	Q5	−0.45 (−0.66, −0.25)	<0.001		−0.05 (−0.18, 0.08)	0.46	
*P for trend*		<0.001	NA		0.47	NA	

Full models were adjusted for age, sex, ethnic background, smoking, education level, Townsend deprivation index, body mass index, history of cardiovascular disease, history of hypertension, history of chronic kidney disease, history of diabetes, lipid-lowering medication use and alcohol consumption frequency. MVX metabolic vulnerability index; MMX, metabolic malnutrition index; IVX, inflammation vulnerability index; PS, photoreceptor segment; RPE–BM, retinal pigment epithelium–Bruch’s membrane; FDR, false discovery rate

## Discussion

In this prospective cohort study, we have, for the first time, demonstrated that the two metabolic metrics, MVX and MetaboHealth, showed a graded positive association with AMD incidence. These associations were stronger among participants with comorbidities, such as CVD and diabetes, than in those without these conditions. Importantly, MVX, characterized by inflammation-related metabolic vulnerability, showed additive and multiplicative interactions with the AMD PRS. Moreover, associations of MVX and MetaboHealth with AMD were further supported by their relationships with PS thickness. These findings collectively highlight the importance of systemic metabolic health in AMD development and suggest that MVX and MetaboHealth are novel biomarkers associated with increased AMD risk.

In recent years, a growing body of evidence from cross-sectional and prospective studies of metabolomics and AMD, as well as from Mendelian randomization (MR) analyses, has indicated a metabolic basis for AMD [8–15, 38]. However, some studies reported inconsistent or null associations [8, 10, 15]. For example, our previous study, together with supporting evidence from a MR analysis, highlighted the role of very-low-density lipoprotein (VLDL) subclasses in AMD risk, but not high-density lipoprotein (HDL) subclasses [8, 10]. In contrast, another study based on five European cohorts indicated an important role of HDL subparticles in early and intermediate AMD [15]. Such discrepancies may reflect differences in metabolite panels, sample size, population characteristics, or differences in AMD stages across the study cohorts. In this study, MVX and MetaboHealth composite scores were associated with an increased risk of incident AMD. MVX was originally developed in cardiovascular populations as a novel marker to quantify systemic inflammation and metabolic malnutrition, and has been validated for its associations with atherosclerosis, heart failure, and mortality [16, 17, 39]. In contrast, the second-generation biological aging biomarker MetaboHealth score has been validated to capture more biologically relevant aging signals than age-trained biomarkers and has been associated with multiple age-related diseases, such as cognitive decline [18, 40, 41]. Therefore, these two metabolic metrics provided a complementary perspective on AMD risk. Our findings also suggest that AMD may share a common cellular aetiology with chronic non-communicable diseases, such as CVD, reinforcing the concept that retinal disease is closely linked to systemic metabolic disturbances [42, 43]. In addition, MetaboHealth led to a modest improvement in model discrimination beyond conventional clinical covariates, whereas MVX did not. These findings suggest that MetaboHealth may provide incremental information beyond established risk factors; however, the magnitude of improvement is small and warrants further validation and evaluation of clinical utility in primary eye care settings.

While the association between MVX/MetaboHealth and AMD was statistically robust, the effect size (e.g., HR per SD = 1.06 for MVX) was modest. AMD has a highly multifactorial nature, and such an association may have limited clinical significance; however, our analysis suggested that several interactions exist. Specifically, MVX showed stronger associations with AMD among participants with CVD than among those without CVD (multiplicative *p* < 0.05). Individuals with CVD have been reported to have higher circulating levels of BCAAs (isoleucine, leucine, and valine), which are main components of MVX and reflect impaired metabolic health [44, 45]. The HDL particle profile, a specific component of MVX, has been linked to CVD risk in previous studies [46, 47]. Another component, GlycA, is known as a biomarker for low-grade chronic inflammation [48]. These biomarker profiles may help explain the observed stronger MVX–AMD association among individuals with CVD, warranting further investigation of the underlying biological mechanisms. In addition, diabetes may represent a pro-aging state, as diabetes-related metabolic disturbances, such as persistent hyperglycaemia and altered lipid metabolism, have been implicated in cellular senescence [49]. Within this framework, we observed a 60% higher AMD risk in the highest quintile of the MetaboHealth score among individuals with diabetes, whereas the corresponding increase was 22% among those without diabetes. Furthermore, chronological age is the dominant risk factor for AMD. We observed MetaboHealth score interacted with age on an additive scale, suggesting joint effects on AMD risk. No significant interaction between MVX and age was observed, indicating similar associations in older and younger individuals, consistent with a previous study on MVX and mortality [16]. These findings highlight the importance of maintaining metabolic health across the life course.

Inflammation and metabolic dysregulation are intimately linked [50], constituting core biological pathways that underlie the pathogenesis of diverse disorders, including AMD. In this context, MVX and IVX, capturing systemic metabolic and inflammatory susceptibility, showed interactions with the AMD PRS, with MVX demonstrating both additive and multiplicative effects (16% of the joint risk attributable to interaction and a combined excess risk 1.58 times greater than the sum of individual effects), whereas IVX showed positive interaction on the multiplicative scale. These two scales of interaction provide complementary perspectives on risk interpretation [51]. Additive interaction quantifies the excess absolute risk due to the coexistence of two exposures and is more relevant from a public health standpoint [52], while multiplicative interaction assesses the deviation from independent effects on a relative scale. Furthermore, effect modification by key genetic factors showed that the associations of MVX and IVX with AMD risk varied by the genotypes of *CFH* rs1061170 and rs10922109, indicating that the observed associations may vary according to complement-related genetic susceptibility.

The retina is a highly nutrient- and oxygen-demanding tissue. The metabolic basis underlying retinal layers has been investigated before [37, 53]. In line with AMD findings, higher MVX was associated with a thinner PS layer. The outer layer of the retina is sensitive to systemic metabolic dysfunction [53, 54]. Systemic metabolic and inflammatory burden, as reflected by metabolic syndrome, has been associated with a thinner PS layer [55]. In addition, as age increases, the PS layer becomes thinner [20], and MetaboHealth score, which captures biological aging, demonstrated an inverse association with the PS layer. The associations were directionally consistent across PS sublayers and remained robust after further adjustment for SE and IOP, lending additional support to our primary findings.

In addition to its large sample size and sufficient covariate adjustments, this study is the first to demonstrate the relevance of MVX and MetaboHealth to AMD incidence, thereby extending these metabolic metrics from cardiovascular and mortality outcomes to ophthalmic conditions. The integration of clinical, genetic and retinal imaging data adds a multidimensional perspective that enhances the robustness of the findings. However, several limitations should be considered. First, as UKB participants were predominantly of European descent, the generalizability of our findings may be limited. Although sensitivity analyses showed consistent results, validation in an independent AMD cohort could strengthen reproducibility. In addition, differences in the genetic architecture of AMD and the distribution of metabolomic biomarkers across ancestries may contribute to heterogeneity in effect estimates; therefore, validation in independent and more ethnically diverse cohorts is warranted. Second, we lacked data on AMD subtypes, limiting our ability to evaluate more refined associations between these composite metrics and different AMD stages. Third, most metabolites were measured only at baseline, precluding an analysis of their longitudinal trajectories. Future research with multiple time-point metabolomics measurements is needed to characterize long-term metabolic trajectories and examine their associations with AMD risk while accounting for time-varying confounding, which may provide further insight into the relative roles of long-term metabolic dysfunction and chronological age. Fourth, the interaction analyses were exploratory and hypothesis-generating in nature, and we did not apply formal multiple-testing corrections. Accordingly, the possibility of type I errors cannot be ruled out, and these interaction findings should be interpreted with caution and warrant validation in independent cohorts before they can inform precision medicine strategies or definitive biological conclusions. Fifth, as with all observational studies, residual confounding cannot be entirely excluded, despite adjusting for key covariates. In addition, the observational nature of this study does not allow causal inferences regarding these metrics. Further experimental studies are warranted to clarify the causal association of MVX and MetaboHealth with AMD and investigate the underlying mechanisms.

## Conclusions

In a large population-based cohort, this study demonstrated that MVX and MetaboHealth were associated with AMD risk, especially in those with comorbidities, such as CVD or diabetes. Further validation of these findings in independent cohorts is warranted before meaningful clinical recommendation can be made.

## Electronic supplementary material

Below is the link to the electronic supplementary material.

Supplementary material 1

## Data Availability

The data analyzed in this study are available from the UK Biobank by application to the resource (http://www.ukbiobank.ac.uk). Access was granted under Application 91,320 following approval of a material transfer agreement.

## Abbreviation

AMD: Age-related macular degeneration
MVX: Metabolic vulnerability index
IVX: Inflammation vulnerability index
MMX: Metabolic malnutrition index
PRS: Polygenic risk score
RPE-BM: Retinal pigment epithelium-Bruch’s membrane
PS: Photoreceptor segment
UKB: UK biobank
HES: Hospital Episode Statistics
ICD: International Classification of Diseases
OCT: Optical coherence tomography
NMR: Nuclear magnetic resonance
CPH: Cox proportional hazards
HR: Hazard ratio
CI: Confidence interval
SD: Standard deviation
CVD: Cardiovascular disease
CKD: Chronic kidney disease
BMI: Body mass index
GlycA: Glycoprotein acetyls
sHDL: Small high-density lipoprotein particles
VLDL: Very-low-density lipoprotein
BCAAs: Branched-chain amino acids
SE: Spherical equivalent
IOP: Intraocular pressure
*CFH*: Complement factor H
*ARMS2*: Age-related maculopathy susceptibility 2
RERI: Relative excess risk due to interaction
AP: Attributable proportion
SI: Synergy index
