# Supplementary material for: Metabolic vulnerability index and MetaboHealth score as risk factors for age-related macular degeneration in a large-scale prospective cohort
Source: J Transl Med. 2026 Feb 18;24:415. doi: 10.1186/s12967-026-07863-2 (PMC13020238; doi:10.1186/s12967-026-07863-2)
Supplement: Supplementary file 1 — Supplementary material 1 [file 12967_2026_7863_MOESM1_ESM.docx]

Additional File 1

[Methods 2](#_Toc219315789)

[Figure S1. Spearman correlations matrix 4](#_Toc219315790)

[Figure S2. Flowchart of the retinal layer analysis procedure 5](#_Toc219315791)

[Table S1. Calculations of sex-specific IVX, MMX, and MVX scores 6](#_Toc219315792)

[Table S2. Covariates of disease codes used in this study 7](#_Toc219315793)

[Table S3. Baseline characteristics of participants by MetaboHealth quintiles 8](#_Toc219315794)

[Table S4. Sensitivity analyses of the association between baseline MVX, MMX, IVX, and AMD incidence 10](#_Toc219315795)

[Table S5. Sensitivity analyses of the association between MetaboHealth and AMD incidence 12](#_Toc219315796)

[Table S6. Discriminative performance of predictive models for incident AMD 13](#_Toc219315797)

[Table S7. Subgroup analyses on associations between MVX and AMD incidence 14](#_Toc219315798)

[Table S8. Multiplicative and additive interaction of subgroup analyses on associations between MVX and AMD incidence 15](#_Toc219315799)

[Table S9. Subgroup analyses on associations between MetaboHealth and AMD incidence 16](#_Toc219315800)

[Table S10. Multiplicative and additive interaction of subgroup analyses on associations between MetaboHealth and AMD incidence 17](#_Toc219315801)

[Table S11. Association between IVX, MMX, MVX, and incident AMD, stratified by AMD genetic risk 18](#_Toc219315802)

[Table S12. Association between MetaboHealth and incident AMD, stratified by AMD genetic risk 19](#_Toc219315803)

[Table S13. Baseline characteristics of participants for the retinal layer analyses 20](#_Toc219315804)

[Table S14. Associations of MVX, MMX, IVX, and MetaboHealth with PS sub-layer thickness 21](#_Toc219315805)

[Table S15. Sensitivity analyses of associations between MVX, MMX, IVX, MetaboHealth, and retinal layer thickness 22](#_Toc219315806)

## **Methods**

**Covariates**

Self-reported ethnicity was ascertained and dichotomized as White (British, Irish, or other White backgrounds) and non-White. The non-White group comprised Asian or Asian British (Indian, Pakistani, Bangladeshi, Chinese, or other Asian), Black or Black British (African, Caribbean, or other Black), Mixed (e.g., White and Black Caribbean/African, White and Asian), and other ethnicities. Socio-economic status was assessed using the Townsend Deprivation Index, an area-level composite of unemployment, non-car ownership, non-home ownership, and household overcrowding; higher scores indicate greater deprivation. Smoking status was categorized as never or ever (former/current). Educational attainment, also self-reported, was stratified into four levels: no formal qualifications; secondary school qualifications (GCSEs, O-levels, CSEs); higher secondary or vocational qualifications (A-levels, NVQ, HND, HNC, or equivalent professional certificates); and college or university degree.(1)

In full models, we adjusted for age, sex, ethnicity, smoking status, education, Townsend Deprivation Index, body mass index (BMI, kg/m²), and individual comorbidities, including history of cardiovascular disease (CVD), hypertension, diabetes, and chronic kidney disease (CKD).

**Retinal layer assessment**

The detailed flowchart of the retinal layer analysis procedure is shown in Figure S2.(2) The photoreceptor segment (PS) layer is defined as the sum of the thickness between the external limiting membrane (ELM) and the inner segment/outer segment (IS/OS) junction, and the thickness between the IS/OS junction and the retinal pigment epithelium (RPE).

**Additive interaction**

Additive interaction reflects whether the joint effect of two exposures exceeds the sum of their individual effects. Interactions on the additive scale were assessed by calculating the relative excess risk caused by interaction (RERI), the attributable proportion (AP), and the synergy index (SI).(3, 4) RERI was calculated as (HR11−HR10−HR01)+1, where HR11 is the hazard ratio for both risk factors present, HR10 is the hazard ratio when only the first exposure is present, and HR01 is the hazard ratio when only the second exposure is present. RERI effects were considered significant when 95% CI of RERI did not contain zero (null hypothesis: RERI = 0). The AP is the proportion of the risk due to the interaction in the doubly exposed group (AP=RERI/HR11, null hypothesis: AP = 0). SI can be interpreted as the ratio of an increased risk due to both exposures to the sum of individual increased risks (SI=(HR11​−1)/[(HR10​−1)+(HR01​−1)], null hypothesis: SI = 1).(5, 6)

## **Figure S1. Pearson correlations matrix**

Age; ethnicity; sex; Townsend Deprivation Index; education level; BMI, body mass index; CVD, cardiovascular disease; hypertension; CKD, chronic kidney disease; diabetes; MVX metabolic vulnerability index; MMX, metabolic malnutrition index; IVX, inflammation vulnerability index; sHDL, small high-density lipoprotein particles; GlycA, glycoprotein acetyls; citrate; isoleucine; valine; leucine.

## **Figure S2. Flowchart of the retinal layer analysis procedure**

MVX, metabolic vulnerability index; OCT, optical coherence tomography; ETDRS, Early Treatment Diabetic Retinopathy Study; SE, spherical equivalent; IOP, intraocular pressure.

## **Table S1. Calculations of sex-specific IVX, MMX, and MVX scores**

| **Female** | |
| --- | --- |
| IVX | 9 + (GlycA * -0.000187) + (S-HDLP * -0.3585) + ((GlycA * S-HDLP) * 0.000348) |
| MMX | ((4 + (Leu * -0.03142) + (Leu² * 0.0000893)) * 0.353) +((7 + (Val * -0.03362) + (Val² * 0.0000689)) * 0.684) + (Ileu * 0.00332) + ((1 + (Citr * -0.0072) + (Citr² * 0.0000573)) * 0.7135) |
| MVX | (IVX * 2.27278) + (ln(MMX) * 12.13511) + (IVX * ln(MMX)) * -1.09312 |
| **Male** | |
| IVX | 9 + (GlycA * -0.00437) + (S-HDLP * -0.52307) + ((GlycA * S-HDLP) * 0.000817) |
| MMX | ((4 + (Leu * -0.01594) + (Leu^2^ * 0.0000291)) * 1.076) + ((7 + (Val * -0.0239) + (Val^2^ * 0.00005)) * 0.414) + (Ileu * 0.01265) + ((1 + (Citr * 0.00906) + (Citr^2^ * -0.0000126)) * 0.5881) |
| MVX | (IVX * 3.54601) + (ln(MMX) * 14.41428) + (IVX * ln(MMX)) * -1.43438 |

Abbreviations: Citr = citrate; Ileu = isoleucine; IVX = inflammation vulnerability index; Leu = leucine; MMX = metabolic malnutrition index; MVX = metabolic vulnerability index; S-HDLP = small HDL particle number; Val = valine.

## **Table S2. Covariates of disease codes used in this study**

| **Diseases** | **ICD-9** | **ICD-10** | **Survey data** |
| --- | --- | --- | --- |
| Diabetes | 250 | E10-E14 | 6153(3), 6177(3),2443(1), 2976, 5890,5901, 20002 (code 1220) |
| CVD (7) | 410-414 | I20-25, I60-69 | 20002 (code 1074, 1075, and 1081) |
| Hypertension (7) | 401-405 | I10-I13, I15, O10 | 6153(2), 6177(2), 2966, 20002 (code 1072 and 1065) |
| CKD | 585,5859 | I12.0, I13.1, I13.2, N18 | Category 42 (data field 42026), 20002 (code 1192,1193 and 1194) |

ICD, International Classification of Diseases. CVD, cardiovascular disease including coronary heart disease and stroke. CKD, chronic kidney disease.

## **Table S3. Baseline characteristics of participants by MetaboHealth quintiles**

| **Baseline characteristics** | **Whole cohort** | **Quintile, Q1** | **Quintile, Q2** | **Quintile, Q3** | **Quintile, Q4** | **Quintile, Q5** |
| --- | --- | --- | --- | --- | --- | --- |
| Number | 264,352 | 52,871 | 52,870 | 52,870 | 52,870 | 52,871 |
| Age, years, mean (SD) | 56.50 (8.07) | 54.32 (8.03) | 55.87 (7.98) | 56.70 (7.95) | 57.32 (7.96) | 58.27 (7.89) |
| Female, n (%) | 142,826 (54.0) | 17,156 (32.4) | 25,878 (48.9) | 30,526 (57.7) | 34,155 (64.6) | 35,111 (66.4) |
| White, n (%) | 251,910 (95.3) | 49,963 (94.5) | 50,430 (95.4) | 50,546 (95.6) | 50,599 (95.7) | 50,372 (95.3) |
| TDI, mean (SD) | -1.38 (3.06) | -1.65 (2.91) | -1.59 (2.94) | -1.48 (3.01) | -1.32 (3.09) | -0.87 (3.27) |
| University degree, n (%) | 84,239 (31.9) | 21,215 (40.1) | 18,572 (35.1) | 16,751 (31.7) | 15,005 (28.4) | 12,696 (24.0) |
| Ever smoker, n (%) | 119,819 (45.3%) | 22,136 (41.9%) | 22,922 (43.4%) | 23,595 (44.6%) | 24,411 (46.2%) | 26,755 (50.6%) |
| BMI, mean (SD) | 27.44 (4.78) | 26.61 (3.73) | 26.83 (4.11) | 27.15 (4.47) | 27.64 (4.91) | 28.95 (5.98) |
| CVD history, n (%) | 17,881 (6.8) | 2473 (4.7) | 2868 (5.4) | 3187 (6.0) | 3773 (7.1) | 5580 (10.6) |
| Hypertension history, n (%) | 78,608 (29.7) | 12,301 (23.3) | 13,717 (25.9) | 14,962 (28.3) | 16,425 (31.1) | 21,203 (40.1) |
| CKD history, n (%) | 3636 (1.4) | 322 (0.6) | 468 (0.9) | 566 (1.1) | 781 (1.5) | 1499 (2.8) |
| Diabetes history, n (%) | 14,317 (5.4) | 1249 (2.4) | 1616 (3.1) | 1918 (3.6) | 2669 (5.0) | 6865 (13.0) |
| MetaboHealth, mean (SD) | 0.00 (1.00) | -1.25 (0.4) | -0.53 (0.14) | -0.077 (0.13) | 0.42 (0.16) | 1.45 (0.08) |
| XXL-VLDL-L, µmol/L, mean (SD) | 222.08 (203.58) | 285.14 (239.84) | 230.42 (202.35) | 211.08 (191.48) | 195.07 (182.93) | 188.70 (180.63) |
| S-HDL-L, µmol/L, mean (SD) | 1171.31 (161.07) | 1211.93 (165.68) | 1185.35 (156.66) | 1172.02 (155.06) | 1158.38 (154.82) | 1128.90 (160.94) |
| VLDL-D, nm, mean (SD) | 38.63 (1.26) | 39.17 (1.19) | 38.77 (1.19) | 38.57 (1.21) | 38.40 (1.22) | 38.24 (1.29) |
| PUFA/FA, percent, mean (SD) | 42.24 (3.76) | 42.42 (4.14) | 42.54 (3.77) | 42.42 (3.64) | 42.24 (3.56) | 41.61 (3.60) |
| GlycA, µmol/L, mean (SD) | 811.64 (120.13) | 772.53 (108.95) | 782.95 (108.60) | 798.80 (110.16) | 821.34 (112.20) | 882.56 (127.01) |
| Glucose, µmol/L, mean (SD) | 3691.55 (1172.55) | 3378.87 (687.81) | 3493.17 (704.56) | 3580.53 (757.02) | 3703.13 (859.86) | 4302.03 (2017.48) |
| Isoleucine, µmol/L, mean (SD) | 51.19 (18.22) | 52.87 (19.15) | 50.90 (18.03) | 50.37 (17.97) | 50.31 (17.73) | 51.52 (18.06) |
| Valine, µmol/L, mean (SD) | 210.36 (43.49) | 223.19 (44.56) | 212.21 (41.94) | 207.65 (42.22) | 204.40 (41.93) | 204.33 (43.87) |
| Leucine, µmol/L, mean (SD) | 104.22 (28.90) | 113.16 (30.00) | 105.85 (28.02) | 102.53 (27.91) | 100.20 (27.62) | 99.36 (28.71) |
| Lactate, µmol/L, mean (SD) | 3947.86 (1110.15) | 3969.12 (1031.11) | 3907.32 (1066.43) | 3905.31 (1087.07) | 3920.62 (1131.23) | 4036.90 (1219.76) |
| Histidine, µmol/L, mean (SD) | 65.55 (11.34) | 70.21 (14.30) | 66.97 (10.14) | 65.22 (9.96) | 63.76 (9.84) | 61.62 (9.83) |
| Phenylalanine, µmol/L, mean (SD) | 47.29 (11.43) | 45.01 (9.99) | 46.05 (9.95) | 46.91 (10.26) | 48.00 (10.63) | 50.51 (14.78) |
| Acetoacetate, µmol/L, mean (SD) | 12.98 (12.29) | 9.85 (7.17) | 11.14 (8.61) | 12.32 (9.86) | 13.93 (11.49) | 17.64 (19.10) |
| Albumin, g/l, mean (SD) | 39.37 (3.38) | 41.29 (3.25) | 40.02 (3.03) | 39.35 (3.03) | 38.71 (3.04) | 37.48 (3.26) |

BMI, body mass index; TDI, Townsend Deprivation Index; CVD, cardiovascular disease; CKD, chronic kidney disease; XXL-VLDL-L, total lipids in chylomicrons and extremely large VLDL; S-HDL-L, total lipids in small HDL; VLDL-D, average diameter for VLDL particles; PUFA/FA, polyunsaturated fatty acids to total fatty acids percentage; sHDL, small high-density lipoprotein particles; GlycA, glycoprotein acetyls.

## **Table S4. Sensitivity analyses of the association between baseline MVX, MMX, IVX, and AMD incidence**

|  | **Sensitivity analysis 1**  **(event/n=4897/247,191)** | | **Sensitivity analysis 2**  **(event/n=5509/11,018)** | | **Sensitivity analysis 3**  **(event/n=5359/265,133)** | | **Sensitivity analysis 4**  **(event/n=2687/265,133)** | |
| --- | --- | --- | --- | --- | --- | --- | --- | --- |
|  | **HR (95%CI)** | ***P*** | **HR (95%CI)** | ***P*** | **HR (95%CI)** | ***P*** | **HR (95%CI)** | ***P*** |
| **MVX** |  |  |  |  |  |  |  |  |
| **Per SD** | 1.09 (1.03–1.15) | 0.0019 | 1.03 (1.01–1.06) | 0.0051 | 1.06 (1.03–1.10) | <0.001 | 1.05 (1.00–1.10) | 0.03 |
| **Q1** | 1.00 (ref) | NA | 1.00 (ref) | NA | 1.00 (ref) | NA | 1.00 (ref) | NA |
| **Q2** | 1.09 (0.94–1.26) | 0.24 | 1.08 (0.98–1.18) | 0.11 | 1.09 (0.99–1.19) | 0.089 | 1.10 (0.96–1.25) | 0.17 |
| **Q3** | 1.10 (0.95–1.28) | 0.21 | 1.09 (1.00–1.19) | 0.048 | 1.09 (0.98–1.20) | 0.099 | 1.07 (0.93–1.23) | 0.37 |
| **Q4** | 1.12 (0.95–1.31) | 0.17 | 1.04 (0.96–1.13) | 0.34 | 1.11 (1.00–1.23) | 0.045 | 1.08 (0.94–1.25) | 0.26 |
| **Q5** | 1.20 (1.02–1.41) | 0.031 | 1.11 (1.03–1.20) | 0.0083 | 1.16 (1.04–1.28) | 0.0053 | 1.11 (0.96–1.29) | 0.14 |
| ***P trend*** | 0.046 |  | 0.05 |  | 0.01 |  | 0.25 |  |
| **MMX** |  |  |  |  |  |  |  |  |
| **Per SD** | 1.05 (1.01–1.10) | 0.022 | 1.03 (1.01–1.06) | 0.012 | 1.06 (1.03–1.09) | <0.001 | 1.07 (1.02–1.11) | 0.002 |
| **Q1** | 1.00 (ref) | NA | 1.00 (ref) | NA | 1.00 (ref) | NA | 1.00 (ref) | NA |
| **Q2** | 1.22 (1.08–1.39) | 0.0017 | 1.08 (1.00–1.17) | 0.065 | 1.09 (1.00–1.18) | 0.039 | 1.19 (1.06–1.34) | 0.0036 |
| **Q3** | 1.18 (1.02–1.37) | 0.029 | 1.04 (0.96–1.12) | 0.39 | 1.08 (0.98–1.19) | 0.142 | 1.19 (1.03–1.36) | 0.016 |
| **Q4** | 1.13 (0.97–1.32) | 0.1038 | 1.10 (1.02–1.19) | 0.013 | 1.14 (1.03–1.25) | 0.011 | 1.17 (1.01–1.34) | 0.031 |
| **Q5** | 1.22 (1.06–1.41) | 0.0052 | 1.10 (1.02–1.19) | 0.017 | 1.20 (1.09–1.31) | <0.001 | 1.24 (1.09–1.42) | <0.001 |
| ***P trend*** | 0.046 |  | 0.013 |  | <0.001 |  | 0.006 |  |
| **IVX** |  |  |  |  |  |  |  |  |
| **Per SD** | 1.07 (1.01–1.14) | 0.027 | 1.02 (1.00–1.04) | 0.098 | 1.04 (1.00–1.08) | 0.041 | 1.02 (0.97–1.08) | 0.39 |
| **Q1** | 1.00 (ref) | NA | 1.00 (ref) | NA | 1.00 (ref) | NA | 1.00 (ref) | NA |
| **Q2** | 0.94 (0.81–1.09) | 0.44 | 0.99 (0.91–1.09) | 0.87 | 1.01 (0.92–1.11) | 0.815 | 0.97 (0.84–1.10) | 0.608 |
| **Q3** | 1.01 (0.85–1.18) | 0.94 | 1.01 (0.93–1.10) | 0.814 | 1.06 (0.96–1.18) | 0.252 | 0.97 (0.84–1.12) | 0.684 |
| **Q4** | 1.04 (0.88–1.24) | 0.66 | 1.03 (0.96–1.11) | 0.42 | 1.06 (0.95–1.19) | 0.265 | 0.99 (0.85–1.15) | 0.852 |
| **Q5** | 1.04 (0.86–1.25) | 0.68 | 1.03 (0.95–1.10) | 0.50 | 1.09 (0.97–1.22) | 0.148 | 0.97 (0.83–1.13) | 0.686 |
| ***P trend*** | 0.43 |  | 0.25 |  | 0.13 |  | 0.81 |  |

Full models adjusted for: age, sex, ethnic background, smoking, education level, Townsend deprivation index, body mass index, history of cardiovascular disease, history of hypertension, history of chronic kidney disease, and history of diabetes. Sensitivity analysis 1. Further adjustment for HDL, total cholesterol, triglycerides, and lipid-lowering medication. Sensitivity analysis 2. Age- and sex-matched propensity score analysis (1:1, caliper = 0.01). This sensitivity analysis controlled for all covariates other than age and sex. Sensitivity analysis 3. Restriction of AMD outcome definition to ICD codes only. Sensitivity analysis 4. Application of a 10-year follow-up window to test stability of associations. MVX metabolic vulnerability index; MMX, metabolic malnutrition index; IVX, inflammation vulnerability index; HR, hazard ratio; SD, standard deviation.

## **Table S5. Sensitivity analyses of the association between MetaboHealth and AMD incidence**

|  | **Sensitivity analysis 1**  **(event/n=4779/230,830)** | | **Sensitivity analysis 2**  **(event/n=5489/10,978)** | | **Sensitivity analysis 3**  **(event/n=5340/264,352)** | | **Sensitivity analysis 4**  **(event/n=2674/264,352)** | |
| --- | --- | --- | --- | --- | --- | --- | --- | --- |
|  | **HR (95%CI)** | ***P*** | **HR (95%CI)** | ***P*** | **HR (95%CI)** | ***P*** | **HR (95%CI)** | ***P*** |
| **MetaboHealth** | |  |  |  |  |  |  |  |
| **Per SD** | 1.15 (1.11–1.18) | <0.001 | 1.08 (1.05–1.11) | <0.001 | 1.14 (1.11–1.17) | <0.001 | 1.15 (1.11–1.20) | <0.001 |
| **Q1** | 1.00 (ref) | NA | 1.00 (ref) | NA | 1.00 (ref) | NA | 1.00 (ref) | NA |
| **Q2** | 1.01 (0.91–1.13) | 0.80 | 0.98 (0.89–1.08) | 0.66 | 1.01 (0.91–1.11) | 0.89 | 1.01 (0.88–1.17) | 0.85 |
| **Q3** | 1.10 (0.99–1.21) | 0.083 | 1.03 (0.94–1.13) | 0.52 | 1.09 (0.99–1.20) | 0.089 | 1.09 (0.95–1.25) | 0.21 |
| **Q4** | 1.15 (1.04–1.27) | 0.007 | 1.08 (0.99–1.18) | 0.087 | 1.15 (1.04–1.26) | 0.005 | 1.10 (0.96–1.25) | 0.19 |
| **Q5** | 1.31 (1.18–1.45) | <0.001 | 1.17 (1.07–1.28) | <0.001 | 1.28 (1.17–1.41) | <0.001 | 1.29 (1.13–1.47) | <0.001 |

Full models adjusted for: age, sex, ethnic background, smoking, education level, Townsend deprivation index, body mass index, history of cardiovascular disease, history of hypertension, history of chronic kidney disease, and history of diabetes. Sensitivity analysis 1. Further adjustment for HDL, total cholesterol, triglycerides, and lipid-lowering medication. Sensitivity analysis 2. Age- and sex-matched propensity score analysis (1:1, caliper = 0.01). This sensitivity analysis controlled for all covariates other than age and sex. Sensitivity analysis 3. Restriction of AMD outcome definition to ICD codes only. Sensitivity analysis 4. Application of a 10-year follow-up window. HR, hazard ratio; SD, standard deviation.

## **Table S6. Discriminative performance of predictive models for incident AMD**

| **Metabolic metrics** | **C index (95% CI)** | **Compared to** | **ΔC (95% CI)** | ***P*** |
| --- | --- | --- | --- | --- |
| **MVX** |  |  |  |  |
| Conventional factors only | 0.7503 (0.7446–0.7560) | NA | NA | NA |
| Combined | 0.7506 (0.7449–0.7563) | Conventional factors only | 0.0003 (-0.001–0.0007) | 0.16 |
| **MetaboHealth score** |  |  |  |  |
| Conventional factors only | 0.750 (0.744–0.756) | NA | NA | NA |
| Combined | 0.752 (0.747–0.758) | Conventional factors only | 0.0022 (0.0012–0.0031) | <0.001 |

Conventional factors included age, sex, ethnic background, smoking, education level, Townsend deprivation index, body mass index, history of cardiovascular disease, history of hypertension, history of chronic kidney disease, and history of diabetes.

## **Table S7. Subgroup analyses on associations between MVX and AMD incidence**

| **Subgroup** | **AMD event/number (%)** | **HR per 1 SD (95%CI)** | ***P*** | **HR Q5 *vs* Q1 (95%CI)** | ***P*** |
| --- | --- | --- | --- | --- | --- |
| **Age, year** |  |  |  |  |  |
| >median | 4486/124,849 (3.6%) | 1.07 (1.03–1.11) | <0.001 | 1.17 (1.04–1.31) | 0.0078 |
| ≤median | 1023/140,284 (0.7%) | 1.07 (1.00–1.15) | 0.054 | 1.13 (0.90–1.43) | 0.29 |
| **Smoking status** |  |  |  |  |  |
| Ever | 2761/120,178 (2.3%) | 1.06 (1.02–1.11) | 0.003 | 1.18 (1.03–1.34) | 0.01 |
| Never | 2748/144,955 (1.9%) | 1.06 (1.01–1.11) | 0.018 | 1.16 (1.02–1.32) | 0.02 |
| **CVD history** |  |  |  |  |  |
| Yes | 612/17,942 (3.4%) | 1.16 (1.06–1.27) | 0.0016 | 1.45 (1.08–1.94) | 0.015 |
| No | 4897/247,191 (2.0%) | 1.05 (1.01–1.08) | 0.008 | 1.12 (1.00–1.25) | 0.044 |
| **Hypertension history** |  |  |  |  |  |
| Yes | 2299/78,867 (2.9%) | 1.08 (1.03–1.13) | 0.002 | 1.18 (1.01–1.38) | 0.039 |
| No | 3210/186,266 (1.7%) | 1.05 (1.01–1.09) | 0.02 | 1.15 (1.00–1.31) | 0.048 |
| **CKD history** |  |  |  |  |  |
| Yes | 137/3673 (3.7%) | 1.24 (1.02–1.50) | 0.03 | 1.80 (0.93–3.47) | 0.08 |
| No | 5372/261,460 (2.1%) | 1.06 (1.02–1.09) | <0.001 | 1.15 (1.03–1.27) | 0.01 |
| **Diabetes history** |  |  |  |  |  |
| Yes | 523/14,370 (3.6%) | 1.11 (1.01–1.22) | 0.03 | 1.27 (0.93–1.72) | 0.13 |
| No | 4986/250,763 (2.0%) | 1.06 (1.02–1.09) | 0.0015 | 1.15 (1.03–1.28) | 0.013 |

Full models adjusted for: age, sex, ethnic background, smoking, education level, Townsend deprivation index, body mass index, history of cardiovascular disease, history of hypertension, history of chronic kidney disease, and history of diabetes. MVX metabolic vulnerability index; HR, hazard ratio; SD, standard deviation.

## **Table S8. Multiplicative and additive interaction of subgroup analyses on associations between MVX and AMD incidence**

|  | **Subgroup** | **Multiplicative *P*** | **RERI** | **AP** | **Synergy Index** |
| --- | --- | --- | --- | --- | --- |
| **Age, year** | >median | 0.73 | 0.20 (-0.16, 0.56) | 0.04 (-0.03, 0.10) | 1.05 (0.96, 1.14) |
|  | ≤median |  |  |  |  |
| **Smoking status** | ever | 0.53 | -0.02 (-0.14, 0.10) | -0.02 (-0.12, 0.08) | 0.91 (0.41, 1.41) |
|  | never |  |  |  |  |
| **CVD history** | yes | 0.018 | 0.19 (-0.00, 0.38) | 0.15 (0.01, 0.29) | 3.34 (-2.89, 9.58) |
|  | no |  |  |  |  |
| **Hypertension history** | yes | 0.66 | -0.03 (-0.15, 0.09) | -0.02 (-0.13, 0.08) | 0.87 (0.36, 1.37) |
|  | no |  |  |  |  |
| **CKD history** | yes | 0.51 | -0.10 (-0.53, 0.33) | -0.08 (-0.44, 0.28) | 0.69 (-0.40, 1.77) |
|  | no |  |  |  |  |
| **Diabetes history** | yes | 0.96 | 0.07 (-0.19, 0.33) | 0.04 (-0.12, 0.21) | 1.14 (0.55, 1.73) |
|  | no |  |  |  |  |

Full models adjusted for: age, sex, ethnic background, smoking, education level, Townsend deprivation index, body mass index, history of cardiovascular disease, history of hypertension, history of chronic kidney disease, and history of diabetes. MVX metabolic vulnerability index; HR, hazard ratio; SD, standard deviation; RERI, relative excess risk due to interaction; AP, attributable proportion due to interaction; SI, synergy index; TDI, Townsend deprivation index; BMI, body mass index.

## **Table S9. Subgroup analyses on associations between MetaboHealth and AMD incidence**

| **Subgroup** | **AMD event/number (%)** | **HR per 1 SD (95%CI)** | ***P*** | **HR Q5 *vs* Q1 (95%CI)** | ***P*** |
| --- | --- | --- | --- | --- | --- |
| **Age, years** |  |  |  |  |  |
| >median | 4469/124,484 (3.6%) | 1.14 (1.10–1.17) | <0.001 | 1.35 (1.22–1.49) | <0.001 |
| ≤median | 1020/139,868 (0.7%) | 1.14 (1.06–1.22) | <0.001 | 1.21 (0.96–1.51) | 0.12 |
| **Smoking status** |  |  |  |  |  |
| Ever | 2752/119,819 (2.3%) | 1.13 (1.09–1.18) | <0.001 | 1.27 (1.11–1.44) | <0.001 |
| Never | 2737/144,533 (1.9%) | 1.15 (1.11–1.20) | <0.001 | 1.29 (1.13–1.47) | <0.001 |
| **CVD history** |  |  |  |  |  |
| Yes | 612/17,881 (3.4%) | 1.16 (1.08–1.25) | <0.001 | 1.33 (0.99–1.79) | 0.058 |
| No | 4877/246,471 (2.0%) | 1.14 (1.11–1.17) | <0.001 | 1.26 (1.14–1.39) | <0.001 |
| **Hypertension history** |  |  |  |  |  |
| Yes | 2287/78,608 (2.9%) | 1.18 (1.14–1.23) | <0.001 | 1.34 (1.15–1.56) | <0.001 |
| No | 3202/185,744 (1.7%) | 1.10 (1.06–1.15) | <0.001 | 1.22 (1.09–1.38) | <0.001 |
| **CKD history** |  |  |  |  |  |
| Yes | 133/3636 (3.7%) | 1.15 (1.00–1.32) | 0.048 | 1.61 (0.72–3.60) | 0.22 |
| No | 5356/260,716 (2.1%) | 1.14 (1.11–1.17) | <0.001 | 1.26 (1.15–1.38) | <0.001 |
| **Diabetes history** |  |  |  |  |  |
| Yes | 519/14,317 (3.6%) | 1.23 (1.16–1.30) | <0.001 | 1.60 (1.11–2.30) | 0.01 |
| No | 4970/250,035 (2.0%) | 1.11 (1.08–1.15) | <0.001 | 1.22 (1.10–1.34) | <0.001 |

Full models adjusted for: age, sex, ethnic background, smoking, education level, Townsend deprivation index, body mass index, history of cardiovascular disease, history of hypertension, history of chronic kidney disease, and history of diabetes. HR, hazard ratio; SD, standard deviation.

## **Table S10. Multiplicative and additive interaction of subgroup analyses on associations between MetaboHealth and AMD incidence**

|  | **Subgroup** | **Multiplicative *P*** | **RERI** | **AP** | **Synergy Index** |
| --- | --- | --- | --- | --- | --- |
| **Age, year** | >median | 0.05 | 0.81 (0.45, 1.17) | 0.13 (0.07, 0.19) | 1.19 (1.09, 1.28) |
|  | ≤median |  |  |  |  |
| **Smoking status** | ever | 0.50 | -0.03 (-0.15, 0.09) | -0.02 (-0.12, 0.07) | 0.90 (0.54, 1.26) |
|  | never |  |  |  |  |
| **CVD history** | yes | 0.44 | 0.10 (-0.10, 0.30) | 0.08 (-0.08, 0.23) | 1.53 (0.02, 3.04) |
|  | no |  |  |  |  |
| **Hypertension history** | yes | 0.11 | 0.08 (-0.04, 0.20) | 0.07 (-0.03, 0.16) | 1.49 (0.40, 2.58) |
|  | no |  |  |  |  |
| **CKD history** | yes | 0.40 | 0.27 (-0.18, 0.72) | 0.20 (-0.11, 0.51) | 4.40 (-16.69, 25.49) |
|  | no |  |  |  |  |
| **Diabetes history** | yes | 0.019 | 0.32 (0.04, 0.60) | 0.20 (0.03, 0.36) | 2.05 (0.33, 3.78) |
|  | no |  |  |  |  |

Full models adjusted for: age, sex, ethnic background, smoking, education level, Townsend deprivation index, body mass index, history of cardiovascular disease, history of hypertension, history of chronic kidney disease, and history of diabetes. HR, hazard ratio; SD, standard deviation. RERI, relative excess risk due to interaction; AP, attributable proportion due to interaction; SI, synergy index; TDI, Townsend deprivation index; BMI, body mass index.

## **Table S11. Association between IVX, MMX, MVX, and incident AMD, stratified by AMD genetic risk**

| **Genotype** | **IVX** | |  | **MMX** | |  | **MVX** |  |  |
| --- | --- | --- | --- | --- | --- | --- | --- | --- | --- |
|  | **HR (95% CI)** | ***P*** | **Multiplicative**  ***P*** | **HR (95% CI)** | ***P*** | **Multiplicative**  ***P*** | **HR (95% CI)** | ***P*** | **Multiplicative**  ***P*** |
| ***CFH***rs10922109 |  |  | 0.014 |  |  | 0.51 |  |  | 0.029 |
| AA | 0.98 (0.88–1.09) | 0.69 |  | 1.06 (0.97–1.15) | 0.200 |  | 1.02 (0.93–1.12) | 0.7 |  |
| AC | 1.02 (0.96–1.08) | 0.54 |  | 1.05 (1.01–1.10) | 0.026 |  | 1.04 (0.99–1.10) | 0.1 |  |
| CC | 1.06 (1.00–1.12) | 0.032 |  | 1.06 (1.02–1.11) | 0.008 |  | 1.08 (1.03–1.13) | 0.0017 |  |
| ***CFH*** rs1061170 |  |  | 0.016 |  |  | 0.90 |  |  | 0.017 |
| TT | 0.95 (0.89–1.01) | 0.099 |  | 1.06 (1.01–1.12) | 0.029 |  | 0.99 (0.93–1.05) | 0.62 |  |
| TC | 1.07 (1.01–1.12) | 0.016 |  | 1.05 (1.01–1.09) | 0.028 |  | 1.08 (1.03–1.14) | 0.00084 |  |
| CC | 1.08 (1.00–1.16) | 0.048 |  | 1.07 (1.00–1.14) | 0.037 |  | 1.10 (1.02–1.17) | 0.0078 |  |
| ***ARMS2*** rs3750846 |  |  | 0.53 |  |  | 0.54 |  |  | 0.42 |
| TT | 1.08 (1.03–1.13) | 0.001 |  | 1.04 (1.00–1.09) | 0.039 |  | 1.09 (1.05–1.14) | 6.16E-05 |  |
| TC | 0.99 (0.93–1.05) | 0.76 |  | 1.08 (1.03–1.13) | 0.002 |  | 1.03 (0.98–1.09) | 0.27 |  |
| CC | 0.91 (0.81–1.02) | 0.13 |  | 1.03 (0.94–1.12) | 0.575 |  | 0.94 (0.85–1.05) | 0.31 |  |
| ***ARMS2*** rs10490924 |  |  | 0.58 |  |  | 0.52 |  |  | 0.45 |
| GG | 1.08 (1.03–1.13) | 0.001 |  | 1.04 (1.00–1.09) | 0.041 |  | 1.09 (1.04–1.14) | 6.64E-05 |  |
| GT | 0.99 (0.93–1.05) | 0.80 |  | 1.08 (1.03–1.13) | 0.002 |  | 1.03 (0.98–1.09) | 0.25 |  |
| TT | 0.91 (0.81–1.02) | 0.13 |  | 1.03 (0.94–1.12) | 0.589 |  | 0.95 (0.85–1.05) | 0.31 |  |

Full models adjusted for: age, sex, smoking, education level, Townsend deprivation index, body mass index, history of cardiovascular disease, history of hypertension, history of chronic kidney disease, and history of diabetes. *P* interaction indicates the test for interaction on the multiplicative scale. IVX, inflammation vulnerability index; MMX, metabolic malnutrition index; MVX metabolic vulnerability index.

## **Table S12. Association between MetaboHealth and incident AMD, stratified by AMD genetic risk**

| **Gene** | **Genotype** | **MetaboHealth** | |  |
| --- | --- | --- | --- | --- |
|  |  | **HR (95% CI)** | ***P*** | **Multiplicative**  ***P*** |
| ***CFH*** | rs10922109 |  |  | 0.47 |
|  | AA | 1.39 (1.15–1.68) | <0.001 |  |
|  | AC | 1.38 (1.25–1.52) | <0.001 |  |
|  | CC | 1.33 (1.21–1.46) | <0.001 |  |
| ***CFH*** | rs1061170 |  |  | 0.08 |
|  | TT | 1.25 (1.11–1.41) | <0.001 |  |
|  | TC | 1.41 (1.29–1.55) | <0.001 |  |
|  | CC | 1.38 (1.22–1.56) | <0.001 |  |
| ***ARMS2*** | rs3750846 |  |  | 0.65 |
|  | TT | 1.38 (1.27–1.50) | <0.001 |  |
|  | TC | 1.34 (1.20–1.49) | <0.001 |  |
|  | CC | 1.35 (1.08–1.68) | 0.0072 |  |
| ***ARMS2*** | rs10490924 |  |  | 0.59 |
|  | GG | 1.38 (1.26–1.50) | <0.001 |  |
|  | GT | 1.34 (1.20–1.50) | <0.001 |  |
|  | TT | 1.35 (1.09–1.68) | 0.0064 |  |

Full models adjusted for: age, sex, smoking, education level, Townsend deprivation index, body mass index, history of cardiovascular disease, history of hypertension, history of chronic kidney disease, and history of diabetes. *P* interaction indicates the test for interaction on the multiplicative scale.

## **Table S13. Baseline characteristics of participants for the retinal layer analyses**

| **Baseline characteristics** | **MVX** | **MetaboHealth** |
| --- | --- | --- |
| Number | 19,422 | 19,377 |
| Age, years, mean (SD) | 55.78 (8.16) | 55.78 (8.16) |
| Female, n (%) | 10,220 (52.6) | 10,193 (52.6) |
| White, n (%) | 18,110 (93.2) | 18,072 (93.3) |
| TDI, mean (SD) | -1.35 (2.89) | -1.35 (2.89) |
| University degree, n (%) | 6737 (34.7) | 6724 (34.7) |
| Ever smoker, n (%) | 8623 (44.4) | 8594 (44.4) |
| BMI, mean (SD) | 27.32 (4.66) | 27.31 (4.66) |
| CVD history, n (%) | 961 (4.9) | 956 (4.9) |
| Hypertension history, n (%) | 5262 (27.1) | 5249 (27.1) |
| CKD history, n (%) | 340 (1.8) | 338 (1.7) |
| Diabetes history, n (%) | 916 (4.7) | 913 (4.7) |
| Lower lipids history, n (%) | 1193 (6.1) | 1189 (6.1) |
| Alcohol frequency, at least once per month n (%) | 3544 (18.2) | 3537 (18.3) |

BMI, body mass index; TDI, Townsend Deprivation Index; CVD, cardiovascular disease; CKD, chronic kidney disease. MVX metabolic vulnerability index.

## **Table S14. Associations of MVX, MMX, IVX, and MetaboHealth with PS sub-layer thickness**

| **Metric** |  | **ELM-ISOS thickness** | |  | **ISOS-RPE thickness** | | |  |
| --- | --- | --- | --- | --- | --- | --- | --- | --- |
|  |  | **Beta (95% CI)** | ***P*** | **FDR *P*** | **Beta (95% CI)** | ***P*** | **FDR *P*** | |
| **MVX** | Per SD | -0.07 (-0.10, -0.04) | <0.001 | <0.001 | -0.05 (-0.12, 0.02) | 0.19 | 0.25 | |
|  | Q1 | ref | NA |  | ref | NA |  | |
|  | Q2 | -0.06 (-0.13, 0.01) | 0.113 |  | 0.11 (-0.07, 0.28) | 0.242 |  | |
|  | Q3 | -0.18 (-0.26, -0.10) | <0.001 |  | 0.03 (-0.16, 0.22) | 0.754 |  | |
|  | Q4 | -0.10 (-0.19, -0.02) | 0.014 |  | 0.03 (-0.17, 0.23) | 0.793 |  | |
|  | Q5 | -0.22 (-0.31, -0.14) | <0.001 |  | -0.04 (-0.25, 0.17) | 0.727 |  | |
| ***P for trend*** | | <0.001 | NA |  | 0.53 | NA |  | |
| **MMX** | Per SD | -0.02 (-0.05, 0.01) | 0.126 | 0.20 | 0.11 (-0.07, 0.28) | 0.242 | 0.28 | |
|  | Q1 | ref | NA |  | ref | NA |  | |
|  | Q2 | 0.02 (-0.07, 0.11) | 0.694 |  | -0.02 (-0.24, 0.20) | 0.866 |  | |
|  | Q3 | -0.10 (-0.19, -0.02) | 0.017 |  | 0.17 (-0.03, 0.38) | 0.095 |  | |
|  | Q4 | -0.05 (-0.13, 0.04) | 0.259 |  | -0.02 (-0.22, 0.18) | 0.87 |  | |
|  | Q5 | -0.06 (-0.14, 0.03) | 0.181 |  | 0.01 (-0.18, 0.21) | 0.904 |  | |
| ***P for trend*** | | 0.22 | NA |  | 0.81 | NA |  | |
| **IVX** | Per SD | -0.07 (-0.10, -0.03) | <0.001 | 0.001 | -0.02 (-0.24, 0.20) | 0.866 | 0.87 | |
|  | Q1 | ref | NA |  | ref | NA |  | |
|  | Q2 | -0.07 (-0.14, 0.01) | 0.075 |  | 0.15 (-0.02, 0.33) | 0.088 |  | |
|  | Q3 | -0.16 (-0.24, -0.08) | <0.001 |  | 0.09 (-0.11, 0.29) | 0.367 |  | |
|  | Q4 | -0.13 (-0.22, -0.05) | 0.003 |  | 0.09 (-0.12, 0.30) | 0.408 |  | |
|  | Q5 | -0.21 (-0.31, -0.12) | <0.001 |  | -0.17 (-0.40, 0.06) | 0.153 |  | |
| ***P for trend*** | | <0.001 | NA |  | 0.13 | NA |  | |
| **MetaboHealth** | |  |  |  |  |  |  | |
|  | Per SD | -0.04 (-0.06, -0.01) | 0.007 | 0.014 | -0.17 (-0.23, -0.11) | <0.001 | <0.001 | |
|  | Q1 | ref | NA |  | ref | NA |  | |
|  | Q2 | -0.01 (-0.08, 0.07) | 0.81 |  | 0.10 (-0.08, 0.28) | 0.28 |  | |
|  | Q3 | -0.06 (-0.14, 0.02) | 0.12 |  | -0.17 (-0.35, 0.01) | 0.07 |  | |
|  | Q4 | -0.09 (-0.17, -0.02) | 0.016 |  | -0.28 (-0.46, -0.09) | 0.003 |  | |
| ***P for trend*** | | 0.016 | NA |  | <0.001 | NA |  | |

Full models were adjusted for age, sex, ethnic background, smoking, education level, Townsend deprivation index, body mass index, history of cardiovascular disease, history of hypertension, history of chronic kidney disease, history of diabetes, lipid-lowering medication use and alcohol consumption frequency. PS, photoreceptor segment; ELM, external limiting membrane; IS/OS, inner-segment/outer-segment junction; RPE, retinal pigment epithelium. IVX, inflammation vulnerability index; MMX, metabolic malnutrition index; MVX metabolic vulnerability index; FDR, false discovery rate.

## **Table S15. Sensitivity analyses of associations between MVX, MMX, IVX, MetaboHealth, and retinal layer thickness**

| **Metric** |  | **PS layer thickness** | |  | **RPE-BM complex thickness** | |  | |
| --- | --- | --- | --- | --- | --- | --- | --- | --- |
|  |  | **Beta (95% CI)** | ***P*** | **FDR *P*** | **Beta (95% CI)** | ***P*** | **FDR *P*** | |
| **MVX** |  |  |  |  |  |  |  | |
|  | Per SD | -0.12 (-0.19, -0.04) | 0.002 | 0.005 | -0.05 (-0.09, -0.00) | 0.042 | 0.084 | |
|  | Q1 | ref | NA |  | ref | NA |  | |
|  | Q2 | 0.11 (-0.08, 0.31) | 0.24 |  | -0.07 (-0.19, 0.05) | 0.233 |  | |
|  | Q3 | -0.04 (-0.25, 0.16) | 0.68 |  | -0.07 (-0.19, 0.06) | 0.31 |  | |
|  | Q4 | -0.04 (-0.25, 0.18) | 0.75 |  | -0.15 (-0.28, -0.01) | 0.034 |  | |
|  | Q5 | -0.24 (-0.47, -0.01) | 0.04 |  | -0.17 (-0.31, -0.03) | 0.018 |  | |
| ***P for trend*** | | 0.018 | NA |  | 0.013 | NA |  | |
| **MMX** | Per SD | -0.01 (-0.08, 0.06) | 0.715 | 0.72 | -0.04 (-0.08, 0.00) | 0.079 | 0.126 | |
|  | Q1 | ref | NA |  | ref | NA |  | |
|  | Q2 | 0.12 (-0.11, 0.36) | 0.31 |  | -0.09 (-0.24, 0.06) | 0.224 |  | |
|  | Q3 | 0.12 (-0.10, 0.35) | 0.272 |  | -0.11 (-0.25, 0.03) | 0.124 |  | |
|  | Q4 | -0.00 (-0.22, 0.22) | 0.998 |  | -0.07 (-0.20, 0.07) | 0.319 |  | |
|  | Q5 | -0.01 (-0.22, 0.20) | 0.938 |  | -0.12 (-0.25, 0.01) | 0.072 |  | |
| ***P for trend*** | | 0.56 | NA |  | 0.15 | NA |  | |
| **IVX** | Per SD | -0.13 (-0.21, -0.05) | 0.002 | 0.005 | -0.03 (-0.08, 0.02) | 0.23 | 0.31 | |
|  | Q1 | ref | NA |  | ref | NA |  | |
|  | Q2 | 0.10 (-0.09, 0.29) | 0.318 |  | -0.11 (-0.23, 0.01) | 0.078 |  | |
|  | Q3 | -0.06 (-0.28, 0.15) | 0.562 |  | -0.06 (-0.19, 0.08) | 0.414 |  | |
|  | Q4 | -0.11 (-0.34, 0.13) | 0.371 |  | -0.08 (-0.22, 0.07) | 0.301 |  | |
|  | Q5 | -0.35 (-0.60, -0.10) | 0.005 |  | -0.12 (-0.27, 0.04) | 0.139 |  | |
| ***P for trend*** | | 0.002 | NA |  | 0.27 | NA |  | |
| **MetaboHealth** | |  |  |  |  |  |  | |
|  | Per SD | -0.250 (-0.32, -0.18) | <0.001 | <0.001 | -0.016 (-0.060, 0.029) | 0.49 | 0.56 | |
|  | Q1 | ref | NA |  | ref | NA |  | |
|  | Q2 | 0.041 (-0.15, 0.23) | 0.67 |  | -0.040 (-0.16, 0.08) | 0.51 |  | |
|  | Q3 | -0.26 (-0.45, -0.063) | 0.009 |  | -0.047 (-0.17, 0.073) | 0.44 |  | |
|  | Q4 | -0.33 (-0.53, -0.14) | <0.001 |  | -0.053 (-0.18, 0.070) | 0.40 |  | |
|  | Q5 | -0.58 (-0.79, -0.37) | <0.001 |  | -0.049 (-0.18, 0.08) | 0.46 | |  |
| ***P for trend*** | | <0.001 | NA |  | 0.45 | NA | |  |

Full models were adjusted for age, sex, ethnic background, smoking, education level, Townsend deprivation index, body mass index, history of cardiovascular disease, history of hypertension, history of chronic kidney disease, history of diabetes, lipid-lowering medication use, alcohol consumption frequency, spherical equivalent and intraocular pressure. PS, photoreceptor segment; RPE, retinal pigment epithelium; IVX, inflammation vulnerability index; MMX, metabolic malnutrition index; MVX metabolic vulnerability index; FDR, false discovery rate.

**References**

1. Mutz J, Roscoe CJ, Lewis CM. Exploring health in the UK Biobank: associations with sociodemographic characteristics, psychosocial factors, lifestyle and environmental exposures. BMC Med. 2021;19(1):240.

2. Patel PJ, Foster PJ, Grossi CM, Keane PA, Ko F, Lotery A, et al. Spectral-Domain Optical Coherence Tomography Imaging in 67 321 Adults: Associations with Macular Thickness in the UK Biobank Study. Ophthalmology. 2016;123(4):829-40.

3. Andersson T, Alfredsson L, Källberg H, Zdravkovic S, Ahlbom A. Calculating measures of biological interaction. Eur J Epidemiol. 2005;20(7):575-9.

4. Li R, Chambless L. Test for additive interaction in proportional hazards models. Ann Epidemiol. 2007;17(3):227-36.

5. Jang YJ, Kang C, Myung W, Lim SW, Moon YK, Kim H, et al. Additive interaction of mid- to late-life depression and cerebrovascular disease on the risk of dementia: a nationwide population-based cohort study. Alzheimers Res Ther. 2021;13(1):61.

6. Vart P, Nigatu YT, Jaglan A, van Zon SK, Shafique K. Joint Effect of Hypertension and Elevated Serum Phosphorus on the Risk of Mortality in National Health and Nutrition Examination Survey-III. J Am Heart Assoc. 2015;4(5).

7. Said MA, Verweij N, van der Harst P. Associations of Combined Genetic and Lifestyle Risks With Incident Cardiovascular Disease and Diabetes in the UK Biobank Study. JAMA Cardiol. 2018;3(8):693-702.
